# Supplementary material for: Subcortical-cortical dynamical states of the human brain and their breakdown in stroke
Source: Nat Commun. 2022 Aug 29;13:5069. doi: 10.1038/s41467-022-32304-1 (PMC9424299; doi:10.1038/s41467-022-32304-1)
Supplement: Supplementary file 1 — Supplementary Information [file 41467_2022_32304_MOESM1_ESM.pdf]

Supplementary Information
Subcortical-cortical dynamical states of the human brain and their breakdown in stroke

Chiara Favaretto[1,2,*], Michele Allegra[1,3,4], Gustavo Deco[5,6], Nicholas V. Metcalf[7], Joseph C. Griffis[7], Gordon L. Shulman[7,8], Andrea Brovelli[4], Maurizio Corbetta[1,2,7,8,9,*]

[1]*Padova Neuroscience Center (PNC), University of Padova, via Orus 2/B, 35129, Padova, Italy*
[2]*Department of Neuroscience (DNS), University of Padova, via Giustiniani 2, 35128, Padova, Italy*
[3]*Department of Physics and Astronomy "Galileo Galilei", University of Padova, via Marzolo 8, 35131, Padova, Italy*
[4]*Institut de Neurosciences de la Timone UMR 7289, Aix Marseille Univerité, CNRS, 13005, Marseille. France*
[5]*Center for Brain and Cognition (CBC), Department of Information Technologies and Communications (DTIC), Pompeu Fabra University, Edifici Mercè Rodoreda, Carrer Trias i Fargas 25-27, 08005, Barcelona, Catalonia, Spain*
[6]*Istituició Catalana de Recerca I Estudis Avançats (ICREA), Passeig Lluis Companys 23, 08010, Barcelona, Catalonia, Spain*
[7]*Department of Neurology, Washington University School of Medichine, 660 S. Euclid Ave, St. Louis, MO, 63110*
[8]*Department of Radiology, Washington University School of Medichine, 660 S. Euclid Ave, St. Louis, MO, 63110*
[9]*Venetian Institute of Molucular Medicine (VIMM), Biomedical Foundation, via Orus 2, 35129, Padova, Italy*

Index

- Supplementary Text
- Supplementary Tables
- Supplementary Figures

Supplementary Text

S1 Control analyses

Several control analyses were run insuring that the definition of DFSs was robust in healthy and stroke patients. First, the computation of DFSs is computationally intensive because it requires to track the dynamics of each pairwise link for each subject and for each time point. To reduce the computational complexity, we applied a spatial dimensionality reduction to scale down the number of cortical parcels: from 324 to 90 Regions of Interest (ROIs)[1] (see SI paragraph S2, SI-Figs.3-4, SI-Table 4).

Second, we insured that the topology of DFSs was a reliable representation of the empirical data from which it was derived, i.e., the dynamic FC in each sliding window (SI-Fig.5). We measured the Pearson's correlation coefficient

between each dynamic FC window and the associated DFS. The distribution of the correlation coefficients was evaluated both for all time windows, and for the time windows associated to each DFS, separately in healthy controls and stroke patients at 2 weeks, 3 months, and 12 months. Stroke patients were further divided between those with severe and mild static FC impairment (see SI paragraph S8). All correlation values were positive ranging between 0.1 and 0.75, with a mean around 0.4. The similarity was equivalent for all groups except more severe stroke patients at 2 weeks.

Third, to achieve a comparable number of healthy controls and patients, we used the data from session 1 and 2 in healthy controls as if they came from different subjects. This choice may lead to an underestimation of the inter-subject variability within the control group. We replicated the analysis by averaging across sessions the dynamic parameters (frequency of occurrence (f), average lifespan (ℓ), and transition probability) derived in each subject. The results were the same (SI paragraph S3, SI-Tables 5-6).

Forth, we checked if the selected sliding window duration (60 sec) influenced the results. The results were similar for time windows between 40 and 100 seconds (SI paragraph S4 and SI-Fig.6-7). We concluded that the procedure for DFS identification provides a relatively accurate and computationally efficient description of the underlying dynamic FC. This procedure is robust in both healthy and stroke patients. Some alterations of FC dynamics are apparent sub-acutely in the most severe stroke patients based on static FC.

Fifth, we analyzed the impact of frame censoring on our results, by recomputing dynamic measures (fraction times, dwell times and transition probabilities) with different censoring thresholds (see SI paragraph S5 and SI-Fig.8). Qualitatively, we did not observe any important differences when varying the threshold.

Sixth, we applied a control analysis, to verify that our major results are not strongly tied to the specific number of DFS (K) choice. In SI paragraph S6 we show that the same results can be obtained with a different choice of K ($K = 10$).

Finally, we wanted to verify the impact of choosing a different subcortical parcellation. Therefore, we performed additional analyses with the subcortical parcellation by Tian et al.[2] to verify the robustness of our major results (SI paragraph S7).

S2 Dimensionality Reduction Procedure

Our analysis was implemented onto fMRI data projected on the cortical surface of each subject, divided into the 324 Regions of Interest (ROIs) developed by Gordon and collegues[1], plus 19 subcortical regions. We used this parcellation to relate our results with previous works analyzing the same dataset[3–8]. However, to avoid computational issues, we applied a spatial dimensionality reduction to our fMRI data.

Methods: dimensionality reduction As described in the *Method Section* of the main text, finding dynamical functional states requires to consider at once a very large set of data, such as the dynamics of each pairwise link ($343 \times 342/2 = 58,653$ links) for each subject and for each time point. Thus, due to computational issues, we needed to apply a spatial dimensionality reduction to our fMRI data (SI-Fig. 3A). Since cortical and subcortical parcels come from two different atlases, we kept all the subcortical regions, and we applied the following steps to aggregate groups of cortical parcels. (i) Firstly, networks with less than three ROIs in each hemisphere were merged with the larger network with the smallest spatial distance: the Retrosplenial Temporal Network with the Visual Network, the Sensory Motor-Hand, and the Sensory Motor-Mouth Networks were combined to form the Sensory Motor-Hand-Mouth Network, the Salience Network with the Ventral Attention Network, and the Cingulo Parietal Network with the Default Mode Network. (ii) After that, for each network (left and right hemisphere separately), we applied a hierarchical clustering algorithm with the *Ward* method[9], based on the ROIs coordinates, to cluster the member ROIs to a set of three or four larger parcels (the Silhouette index[10] was used to select three or four clusters). As a result of these two steps, we obtained a new parcellation of the whole brain into 90 parcels (71 cortical, and 19 subcortical). 63 of the 71 cortical regions were divided into 8 networks (Visual Network (VIS), Sensory Motor Hand-Mouth Network (SMN), Auditory Network (AUD), Cingulo Opercular Network (CON), Ventral Attention Network (VAN), Dorsal Attention Network (DAN), Fronto Parietal Network (FPN), Default Mode Network (DMN)), while the remaining 8 regions were not assigned to any networks (None).
Functional connectivity (FC) measure for both reduced and unreduced data was evaluated as the z-Fisher transform of the pairwise Pearson's correlation coefficient (ρ) between each pair of time series.

Methods: Dimensionality reduction Quality Check. The goodness of the spatial dimensionality reduction was evaluated in two steps. (1) In the first step (SI-Fig. 3B), we evaluated the similarity of the reduced FC with the unreduced FC, and we checked that within-network connectivity was significantly stronger

than between-networks connectivity.
To evaluate the similarity between reduced and unreduced FC, we compared results of the reduced and unreduced parcellation at the network level. For each subject separately, we computed a network-wise (8×8) dissimilarity matrix, whose (i, j) entry represents the Kullback-Leibler divergence (KLD)[11] between the distribution of connectivity values obtained on links between i and j in the unreduced and the reduced case (diagonal elements (i, i) represent links internal to network i).
Then, we pooled the KLD values over pairs of networks and subjects and compared the KLD distribution with a null model (see below). Moreover, to verify that the ranking of connectivity (among subjects) has remained invariant, for each subject we computed network-wise average (8×8) connectivity matrices, whose (i, j) entry represents the average connectivity values for links between i and j, for the reduced and unreduced cases. For each pair of networks (i, j), we then computed the Spearman's correlation (over subjects) between the average (i, j) connectivity in the reduced and unreduced cases, obtaining an 8×8 similarity matrix.
The average connectivity for the reduced FC of every subject was used also to test the significant difference between within- and between-networks connectivity. Specifically, for each network, a t-test was run across subjects to verify that the averaged connectivity within the network was significantly different (stronger) than the averaged connectivity between the network and all the others.
To quantify the statistical significance of the KLD and Spearman's correlation, we built a proper null model by assigning randomly the 343 ROIs to the reduced 90 parcels. In such null model, the 90 parcels do not provide a meaningful dimensionality reduction of the original 343 parcels but combine randomly parcels in different parts of the brain. We compared the obtained values of KLD and Spearman's correlation with those obtained in 1000 random instances of the null model. Only values smaller (or larger) than the 2.5th (97.5th) percentile were considered significantly small (or large).
(2) The second step of the quality check was aimed at verifying that all previous results on stroke impairment in static FC were reproduced with the reduced data. Specifically, it is known that stroke causes two main FC abnormalities: a decrease of homotopic connectivity[3,4,12–18], and an increase of between-network intra-hemispheric connectivity (especially between DAN and DMN)[3,4,19,20], which are reflected by an overall decrease of network modularity[3,21]. Therefore, we performed a t-test for each network average homotopic connectivity to test differences between the control groups and the stroke patients at the acute stage. Similarly, a t-test was used to verify impairments in average DAN-DMN intra-hemispheric connectivity. Finally, we measured network modularity (Newman's

Q) using the `community_louvain` function from the *Brain Connectivity Toolbox*[22], using the 63 cortical regions with a priori assignment to specific networks. Indeed, similarly to Siegel and collegues[3] we defined modules a priori and evaluated the modularity on binarized FC matrices, after applying multiple connectivity thresholds ranging from 4% and 50% connectivity density in steps of 4%. The final modularity measure was obtained by averaging over density thresholds.
A t-test between controls' and acute patients' modularity was evaluated both for each threshold and for the final modularity measure.

Results. SI-Fig. 4A shows the centers of mass of the reduced and unreduced ROIs. Based on the first of two criteria described in the previous Subsection, the unreduced and the reduced FC obtained with the two parcellations schemes resulted to be very similar for all conditions (CTRs and PATs at different time points), in terms of Kullback-Leibler divergence (KLD). This measure was evaluated separately for each condition, considering the whole FC matrix and each separate network (SI-Fig. 4C, top; SI-Table 4).
Moreover, the relationship among subjects remained unchanged for each network, as confirmed by the high values of Spearman's correlation coefficients (SI-Fig. 4C, center).
Finally, to verify that the topology of the brain network has remained invariant after the dimensionality reduction step, we performed a t-test to test the significant difference between within- and between-network connectivity, both for each network separately, and the whole reduced FC matrix (SI-Fig. 4C, bottom). In all cases, and for all conditions (CTRs and PATs at different time points), we found a significantly larger within-network than between-network connectivity.
In summary, the dimensionality reduction algorithm did not affect either the main topological characteristics of the FC matrices or the relationship among subjects.

In relation to the second quality check criterion, we checked that this reduced parcellation was sensitive to the changes induced by stroke lesions. As expected from previous work[3,4,6,7,12–18] we confirmed that stroke patients at the acute stage (2 weeks) had lower homotopic connectivity ($p < 10^{-3}$), stronger DAN-DMN intra-hemispheric connectivity ($p < 10^{-3}$), and lower network modularity ($p < 10^{-3}$) than the healthy control population (SI-Fig. 4D).

S3 Analysis of the impact of dealing with control subjects numerosity in Dynamical Functional States frequency of occurrence

To achieve a comparable number of controls and patients, avoiding that one group prevail over the other during clustering analysis, in the main analyses, we used all the controls' data as if they were from different subjects. Aware that this choice may lead to an underestimation of the inter-subject variability within the control group, we replicated the analysis by considering the average of results obtained for the control subjects at both time points.
For each session, the time series of 20 control subjects were suitable for the analysis: 18 of them participated to both sessions, while 4 of them only once, thus yielding to 22 different control subjects in total. We used the CTRs' BOLD signals of both sessions to determine the DFSs (as in the main text), but all the derived dynamical measures (frequency of occurrence, average lifespan and transition probability) were averaged over sections for the subset of control subjects, who participated to both sections.
In SI-Tables 5-7 we reported the dynamical measures obtained through this analysis, together with those in the main text. Moreover, we applied an unpaired t-test for each DFS, to verify that the two analyses yielded to not-significantly different results.
Importantly, all tests are far from significance, thus confirming that our choice of considering all the controls' data as if they were from different subjects did not affect the results.

S4 Control Analysis on the impact of Sliding windows width selection

As largely discussed in the literature, the choice of sliding windows width is a critical point during dynamical functional connectivity analysis[23–25]. Indeed, sliding windows should be neither too short, in order to avoid being noise-affected, nor too long, to allow functional fluctuation to be identified. In our main analysis, we selected a sliding window width of 30 TR (60 s), based on previous results[23]. However, we tested how much our results were sensitive to our choice. Specifically, we considered other three possible different widths (20 TR, 40 TR and 50 TR) and we used the Silhouette and Davies-Bouldin indexes (as in the main text) to evaluate the best K-means-derived number of states. SI-Fig. 6 shows that pattern of Silhouette and Davies-Bouldin indexes for all the three widths are very similar to the pattern obtained in the main text (width of 30 TR). Indeed, in all cases we would select $K = 5$ Dynamical Functional States.
The same number of DFSs do not guarantees that the DFSs evaluated with other sliding windows widths are similar to the DFSs presented in the main analysis. Thus, for each width, we determined the 5 DFSs (see SI-Fig. 7)and

we compared them with the 5 DFSs described in the main text, in terms of Pearson's correlation similarity index (not shown). Each DFS was highly positively correlated with its corresponding DFS obtained with 30 TR, and very low correlated with all the others.
From these results, we can confirm that our choice for the sliding window width did not impact our main results.

S5 Control Analysis on the impact of censoring

We performed control analyses to check the impact of censoring. We recomputed dynamic measures (fraction times, dwell times and transition probabilities) with different censoring thresholds, a much more stringent threshold of 0.25, as well as a more liberal threshold of 0.75 (to facilitate comparison, dynamic measures were computed on the same set of DFS previously identified with a censoring threshold of 0.5). Results are shown in SI-Fig. 8. Qualitatively, we did not observe important differences when varying the threshold. Quantitatively, among the three types of dynamical measures, transition probabilities were more strongly affected by the change in the threshold. However, fraction times were very robust. In particular, the relevant group differences identified in the main text did not depend on the threshold chosen. Refer to SI-Paragraph S8 for the definition of severe and mild patients. To be more precise, with threshold = 0.25 all differences in fraction times between CTRs and severe sub-acute patients (DFS1, DFS2, DFS3, and DFS4), as well as the difference in DFS5 between CTR and mild sub-acute patients still hold, after FDR correction. With threshold = 0.75, we confirmed the significant difference in fraction times for DFS2 and DFS3 between CTRs and severe sub-acute patients.
In addition, for both thresholds (0.25 and 0.75), we verified that our main findings of cortical vs. subcortical reorganization pattern still hold. SI-Fig. 9-10 shows that the results are not affected by a different choice of censoring threshold.

S6 Control Analysis on the number of selected DFSs

In general, several major results of our work not strongly tied to the specific K chose (see SI-Fig.2). For instance, in the main text, using $K = 5$ we observed that severe stroke patients have higher fraction time for dynamical states with low inter-hemispheric integration (such as DFS2 and DFS4). This finding emerges even if a different K is used. This can be well exemplified by looking at results for $K = 5$. The set of DFS for $K = 5$ can not be immediately and trivially related to those for $K = 10$. However we can sort the states for $K = 10$ based on the total homotopic FC (from highest to lowest), as in SI-Fig.11(a).

By looking at fraction times, we observe that severe patients over-express the state(s) with low homotopic FC (SI-Fig.11(b)). This is not true for mild patients. Hence, this feature is robustly observed across different DFS decompositions. Let us further stress that some of our key findings - such as the fact that cortical and subcortical FC shifts are simultaneous - does not depend on the chosen set of DFS.

S7 Control Analysis on the effect of the subcortical parcellation

To verify the impact of choosing a different subcortical parcellation, we performed additional analyses with the subcortical parcellation by Tian et al.[2] and verified the robustness of three main findings: 1) the 'antagonistic' dynamics of basal ganglia vs limbic regions, represented by two anticorrelated principal components of subcortical dynamic FC; 2) the observation that different DFS are associated with different patterns of cortical/subcortical interactions, as shown by different patterns of connectivity between the main subcortical clusters and cortical networks; and, 3) the coordination between cortical and subcortical dynamics, as shown by simultaneous cortical/subcortical FC shifts. Tian et al.[2] provide four subcortical parcellations with increasing levels of resolution (16, 32, 50, or 54 regions respectively). We limited our analysis to the coarsest (16 regions) and the finest (54 regions) parcellations. Results are presented in SI-Fig. 12 and 13, respectively. We recomputed the PCs of the leading eigenvector time courses of subcortical regions in the 16-region parcellation by Tian et al. The first PC, which explains 32% of the variance, loads strongly on basal ganglia (caudate, putamen, globus pallidus) and anterior thalamus, and weakly on posterior thalamus and nucleus accumbens; the second PC, which explains 18% of the variance, loads strongly on limbic regions (hippocampus and amygdala) and weakly on posterior thalamus (SI-Fig. 12(a)). This result broadly agrees with previous findings obtained with the freesurfer parcellation: subcortical regions roughly split into a 'basal ganglia' and a 'limbic' cluster. Qualitatively, the only main difference between results in the two parcellations is related to the thalamus. While in the previous parcellation the thalamus essentially grouped with the basal ganglia, the new parcellation yields a more nuanced picture, hinting at a division between different parts of the thalamus: the anterior portion of the thalamus groups with the basal ganglia, whereas the posterior portion cannot be clearly affiliated to either of the two clusters (basal ganglia/limbic). We then performed an analogous analysis with the 54-region parcellation by Tian et al. The first PC, which explains 31% of the variance, loads strongly on caudate, putamen, anterior globus pallidus, anterior thalamus, and weakly on posterior thalamus, posterior globus pallidus and nucleus accumbens. The second PC, which explains 11% of the variance, loads

strongly on limbic regions (hippocampus and amygdala), posterior thalamus, some parts of the anterior thalamus, and and weakly on other parts of the anterior thalamus (SI-Fig. 13). These results strengthen the picture obtained in the 16-region parcellation, but show finer splits within the thalamus, with some portions of the anterior thalamus being associated with the first PC, others with the second PC. In summary, the results in the two parcellations confirm the split between basal ganglia and limbic regions, yielding the additional insight that the thalamus if functionally subdivided into regions showing differential association with the two main clusters. We checked whether different DFS are associated with different patterns of cortical/subcortical interactions. SI-Fig. 12 shows the average connectivity between the two subcortical PCs and each cortical network in each DFS. As previously discussed, we think that the most important point is not validating the specific DFS found (the number and shape of each DFS can depend on details of the analysis), but rather the global picture of FC dynamics emerging from DFS analysis (including, chiefly, the interplay between cortical and subcortical regions). Therefore, in our new analyses, we did not repeat the clustering step to find new DFS. We only observed whether the old DFSs (obtained using the Freesurfer parcellation of subcortical regions) correspond to different patterns of cortical/subcortical interaction in the new subcortical parcellation by Tian et al. We used the previous assignment to one of five DFSs, and recomputed the connectivity between subcortical PCs and cortical networks in DFSs, using the new subcortical PCs obtained with the parcellation by Tian et al. Results are in qualitative agreement with previous results. Each DFS is characterized by a different set of cortical and cortical-subcortical interactions. Congruently with previous analyses, DFS1 is characterized by a positive correlation between DMN and the limbic cluster, which in turn is negatively correlated with sensory-motor-attention networks. DFS3 is characterized by a negative correlation between DMN and the limbic cluster, which in turn is positively correlated with sensory-motor-attention networks (conversely, the basal ganglia cluster is negatively correlated with DMN and positively correlated with sensorimotor networks). In DFS4 all subcortical regions show positive correlation. Similar evidence is obtained in the 54-region parcellation [not shown]. Finally, we repeated the analysis on the temporal coordination of changes in cortical and subcortical connectivity using the subcortical parcellations by Tian et al. We evaluated connectivity shifts, defined as connectivity differences between pairs of consecutive sliding windows, separately for cortical and subcortical regions. We defined connectivity jumps when a large connectivity difference occurred (0.29, corresponding to the top 5% values), and tested the simultaneity of cortical and subcortical reorganization by comparing the probability that cortical and subcortical jumps occur simultaneously. For all networks, we found that the observed conditioned probability was significantly larger than the probability

under the null hypothesis (all $p < 10^{-4}$, Bonferroni corrected for 9 networks).

S8 Patients divided into two groups based on low or high static FC impairments

Methods To test whether dynamical measures' impairments were related to static FC abnormalities, we needed a compact description of the static FC abnormalities. Thus, we z-scored the acute patients' FC matrices with respect to the averaged control subjects' FC, and we performed a spatial Principal Components Analysis (PCA), to find the abnormalities patterns explaining the largest portion of variance over patients. To define the static principal components we used only the subset of patients that were not employed for the DFSs' definition ($114 - 47 = 67$ subjects). We retained only the static principal components which explained at least 5% of the total variance, and which corresponded to an eigenvalue of the covariance matrix larger than 1, yielding to 2 components, as described in the Results Subsection S8. The scores of the data projected onto these 2 static components' space were then summed up, to obtain a unique static description having the largest correlation (in absolute value) with the three FC measures more related to stroke: homotopic connectivity, DAN-DMN intra-hemispheric connectivity, and network modularity. This static measure will be used in the following.

Then, we projected the z-scored FCs of the patients used for the dynamical analyses onto the space described by the selected static principal components, and their sum was used to test whether the DFSs dynamics were different between patients with positive (more FC abnormalities) and negative (less FC abnormalities) sum of the static pricipal components through nonparametric permutation tests (when pairs of groups were tested), or One-Way Kruskal-Wallis test (for comparisons between more than two groups).

Results Given the heterogeneity in lesion location and behavioral deficits across patients, we know that FC dynamics would be differently affected depending on the severity of static FC impairment. Hence, we applied a Principal Component Analysis (PCA) to the static FC of acute patients (after z-scoring to the average FC of controls subjects) to divide patients into two groups. To avoid biases induced by using the same subjects, we used the 67 acute patients not suitable for the dynamic analysis as input for the static PCA. From this analysis, the first 2 static components representing 13.05% of the total variance (7.02% and 6.03%, respectively) were sufficient to represent the main static FC anomalies occurring in stroke, i.e., a reduction of homotopic connectivity and network modularity, and an increase of DAN-DMN intra-hemispheric connectivity (Fig.3).

Indeed, the data projected onto the space of the sum of these components were strongly anti-correlated with the average of homotopic connectivity (corr=-0.83, p= for patients used for the static PCA; corr=-0.78, p= for patients used for the dynamical analysis) and with network-modularity (corr=-0.76, p= for patients used for the static PCA; corr=-0.62, p= for patients used for the dynamical analysis). They were also strongly correlated with DAN-DMN intra-hemispheric connectivity (corr=0.70 for patients used for the static PCA; corr=0.57 for patients used for the dynamical analysis) (SI-Fig.15(C) scatter plot).

Then, we used the sign of the sum of the data projected onto components 1 and 2 to divide an independent group of patients used for the dynamic FC analysis into two groups: the group with positive sum of scores (n=18) represents patients with more serious static FC impairments, while the group with negative sum (n=29) characterized patients with a mild abnormality in static FC w.r.t. controls. In terms of lesion anatomy, more severe patients had more frequently basal ganglia and central white matter damage associated with the deep middle cerebral artery distribution, which matched the average lesion distribution (SI-Fig.1). Mild patients had lesions that were more cortical and both in the middle and posterior cerebral artery distribution (SI-Fig.15(C)). From the total NIHSS (National Institutes of Health Stroke Scale[26]), through a t-test we verified that patients with positive sum of static principal components' score were overall more severe in their neurological impairment (t=4.02, p=2.610-4). These findings are consistent with previous work showing that subcortical stroke lesions cause more functional disconnection[3,7,8,27], and more severe behavioral impairment than cortical lesions[5,28].

S9 Correlation across dynamic measures and principal components

Methods Principal Components Analysis (PCA) on dynamic measures For each DFS, a set of dynamical measures could be extracted. Specifically, for each subject, a vector of DFSs' dynamics was obtained by concatenating all the defined measures (main text Fig. 2):

$$\left[\underbrace{f_1,\ldots,f_K}_{K},\underbrace{\ell_1,\ldots,\ell_K}_{K},\underbrace{\mathrm{DFS1}{>}2,\ldots,\mathrm{DFS}K-1{>}K}_{K\times(K-1)}\right] \tag{1}$$

which is composed of $K(K+1)$ elements. However, by construction, these measures are not completely uncorrelated, and some of them could be merged. To analyze the relationship among dynamical measures in the healthy condition,

we performed a Principal Component Analysis (PCA) on the dynamical vectors of control subjects, after z-scoring each vector entry over subjects. Only the components which explained at least 10% of the total variance, and which corresponded to an eigenvalue of the covariance matrix larger than 1 were retained, and the data projected onto the space defined by these components could be considered as a synthetic description for all the dynamical measures of control subjects. The loadings of each principal component (PC) identified covariation patterns among the set of dynamical measures, each pattern including both positive and negative covariation between the individual measures. All measures derived from patients' data (at each condition) were z-scored with respect to averaged control measures and were projected onto the same Principal Components-derived space, to obtain the PCs values of each patient at a different recovery stage. Finally, the patients' values of each component were again z-scored w.r.t control subjects, to highlight dynamic PCs abnormalities.

Results By construction, the dynamical measures used to describe the DFS dynamics were intercorrelated. To obtain a more compact index of dynamics, we applied a PCA over all the measures in control subjects (after z-scoring), i.e., 5 frequencies (f_1 - f_5), 5 durations (ℓ_1 - ℓ_5), and 20 transitions (DFS1 - DFS5 in all combinations). Based on the explained variance, and the covariance matrix's eigenvalues, we computed three components (Dyn-PC1: 18.30% explained variance; Dyn-PC2: 14.12%; Dyn-PC3: 10.79%) explaining about 43% of the variability across states. Each dynamic PC loads on a unique combination of DFS frequency (f), lifespan duration (ℓ), and transitions (e.g., DFS1$><$3). The three dynamical PCs (Dyn-PC) nicely summarize the patterns of results on the individual measures. Specifically, Dyn-PC1 loads positively on dynamical measures related to DFS1 whereas it loads negatively on DFS2 and DFS5. Thus, a subject with a high positive Dyn-PC1 score will have a prevalence of segregated DFS1 over integrated DFS 2 and DFS5. Dyn-PC2 loads positively on DFS3, whereas it negatively loads on transitions related to DFS2 and DFS4. Thus, a high positive value in Dyn-PC2 indicates a prevalence of states 3 over states 2 and 4. Finally, PC3 loads mostly positively on DFS4 and transitions between DFS4 and DFS5.

Next, we projected patients' dynamical measures onto the Dyn-PCA space (after z-scoring w.r.t. CTRs), to identify low-dimensional dynamical biomarkers for stroke. We identified a significant interaction between Dyn-PCs and groups of patients ($F = 5.68$, $p < 0.001$). More severe patients showed lower PC2 scores than CTRs ($t = 3.93$, $p = 0.004$, Bonferroni corrected), indicating a prevalence of DFS2 and 4 over DFS3 and 5. This is coherent with the results of the single measures presented above. Low Dyn-PC2 scores in severe patients

normalized over time as the individual measures ($t = 3.15$, $p = 0.05$, Bonferroni corrected) (SI-Fig. 16).

In summary, in relation to the first query of whether stroke causes changes in brain connectivity dynamics, our analyses show that strokes cause an alteration of the probability of dynamic states pushing the networks toward a stronger integration (DFS2,4), especially in severe patients, but that a more balanced segregation/integration recovers over time.

Supplementary Tables

Demographic information of control and patient populations (part 1 of 2)

	controls					patients				
	**ID**	**age**	**gender**	**education**	**handedness**	**ID**	**age**	**gender**	**education**	**handedness**
	02	21	M	12	R	024	48	F	12	R
	03	49	M	12	R	030	50	F	14	R
	04	61	M	14	R	040	66	M	12	R
	06	61	F	12	R	043	47	M	12	R
	07	54	F	14	R	051	56	F	11	R
	09	50	F	12	R	056	52	M	11	R
	10	45	M	13	R	058	53	M	12	R
	11	53	F	15	R	060	56	F	16	R
	12	64	M	16	R	063	45	F	9	R
	14	70	M	20	R	065	50	M	12	R
	21	79	M	14	R	067	54	M	16	R
	24	51	M	12	R	071	64	F	16	R
	25	60	F	12	R	078	62	M	12	R
	26	53	M	14	R	081	57	M	12	R
	27	56	F	14	R	087	57	M	13	R
	28	55	F	11	R	090	52	M	12	R
	29	53	M	18	L	092	66	M	16	R
	30	58	M	13	L	097	44	M	14	R
	32	49	M	12	R	099	54	F	12	R
	33	53	F	13	R	100	42	M	11	R
	34	68	M	16	R	101	58	M	12	L
	36	83	F	16	R	102	38	F	18	R
						103	53	M	14	R
						105	22	F	16	R
						108	70	F	14	R
						109	40	F	16	R
						111	56	M	9	R
						145	40	M	11	R
						155	57	M	16	R
						161	51	M	12	R
						162	52	M	12	R
						164	47	F	18	R
						165	52	M	14	R
						166	60	M	15	L
						168	50	M	13	R
						169	65	M	20	R
						170	57	M	14	R
						173	52	F	13	R
						175	51	M	9	R
						178	40	F	18	R
						181	52	F	11	R
						182	52	F	11	R
						186	60	M	18	R
						190	69	F	12	R
						193	53	F	12	R
**average**		56.6		13.9			52.3		13.4	
**percentage**			M=59% F=41%		R = 91% L=9%			M=57% F=43%		R=94% L=6%

SI-Table 1: Demographic information of control and patient populations. Source data are provided as a Source Data file.

Individual lesion information (part 1 of 2)

ID	lesion etiology	lesion volume	lesion site	lesion overlap cortical GMmask	lesion overlap subcortical GMmask	lesion overlap WMmask
24	ischemic	4233	cortico-subcortical	0.8% mask<br>18.7% lesion	0.2% mask<br>0.5% lesion	7.9% mask<br>79.5% lesion
27	ischemic	7574	cortico-subcortical	1.7% mask<br>23.4% lesion	19.6% mask<br>25.7% lesion	8.5% mask<br>47.6% lesion
30	ischemic	5332	cortical	3.7% mask<br>71.2% lesion	0% mask<br>0% lesion	2.9% mask<br>23.3% lesion
40	other	5997	cortico-subcortical	2.5% mask<br>43.8% lesion	10% mask<br>16.5% lesion	4.6% mask<br>32.7% lesion
43	ischemic	4275	subcortical	0.1% mask<br>2.3% lesion	12.9% mask<br>30% lesion	6.2% mask<br>61.8% lesion
51	ischemic	104	subcortical	0% mask<br>0% lesion	0.1% mask<br>8.7% lesion	0.2% mask<br>90.4% lesion
56	ischemic	1399	cortical	0.5% mask<br>33% lesion	0% mask<br>0% lesion	2.2% mask<br>65.6% lesion
58	ischemic	294	subcortical	0% mask<br>0% lesion	0.4% mask<br>12.6% lesion	0.6% mask<br>86.1% lesion
60	ischemic	371	brainstem	0% mask<br>0% lesion	0.4% mask<br>10.8% lesion	0% mask<br>0% lesion
63	ischemic	5177	cortical	3.7% mask<br>74.3% lesion	0.1% mask<br>0.2% lesion	2.7% mask<br>22.5% lesion
65	ischemic	119	brainstem	0% mask<br>0% lesion	0% mask<br>0% lesion	0% mask<br>0% lesion
67	ischemic	118	white matter only	0% mask<br>0% lesion	0% mask<br>0% lesion	0.3% mask<br>100% lesion
71	ischemic	13	brainstem	0% mask<br>0% lesion	0% mask<br>0% lesion	0% mask<br>0% lesion
78	ischemic	991	cortical	0.2% mask<br>17.8% lesion	0% mask<br>0% lesion	1.9% mask<br>82.2% lesion
81	ischemic	111	subcortical	0% mask<br>0% lesion	0.7% mask<br>64% lesion	0.1% mask<br>36% lesion
87	ischemic	2565	cortical	1.5% mask<br>59.8% lesion	0% mask<br>0% lesion	2.3% mask<br>37.4% lesion
90	ischemic	91	brainstem	0% mask<br>0% lesion	0% mask<br>0% lesion	0% mask<br>0% lesion
92	ischemic	470	cortical	0.2% mask<br>50% lesion	0% mask<br>0% lesion	0.4% mask<br>37% lesion
97	ischemic	771	cortical	0.3% mask<br>42.4% lesion	0% mask<br>0% lesion	1% mask<br>56.2% lesion
99	ischemic	13895	cortico-subcortical	7.6% mask<br>56.8% lesion	6.5% mask<br>4.6% lesion	9.1% mask<br>28% lesion
100	ischemic	5160	cortico-subcortical	2.6% mask<br>51.3% lesion	0.6% mask<br>1.2% lesion	5.5% mask<br>45.7% lesion
101	ischemic	2007	cerebellar	0.1% mask<br>4.8% lesion	0% mask<br>0% lesion	0% mask<br>0% lesion
102	ischemic	162	subcortical	0% mask<br>0% lesion	1.6% mask<br>98.8% lesion	0% mask<br>0% lesion
103	hemorrhagic	11301	cortico-subcortical	2.8% mask<br>25.9% lesion	23.5% mask<br>20.6% lesion	13.4% mask<br>50.4% lesion

SI-Table 2: Individual lesion information (part 1 of 2). GM = gray matter; WM = white matter. Source data are provided as a Source Data file.

Individual lesion information (part 2 of 2)

ID	lesion etiology	lesion volume	lesion site	lesion overlap cortical GMmask	lesion overlap subcortical GMmask	lesion overlap WMmask
105	hemorrhagic	4020	cortico-subcortical	2.1% mask<br>54.4% lesion	0.3% mask<br>0.7% lesion	4% mask<br>42.6% lesion
108	ischemic	6287	cortical	4.2% mask<br>69.5% lesion	0% mask<br>0% lesion	3.4% mask<br>23.3% lesion
109	hemorrhagic	14768	cortico-subcortical	8.5% mask<br>59.2% lesion	4.3% mask<br>2.9% lesion	11.8% mask<br>34.1% lesion
111	ischemic	1718	white matter only	0.1% mask<br>4.1% lesion	0% mask<br>0% lesion	3.9% mask<br>95.8% lesion
140	other	10300	cortico-subcortical	2.7% mask<br>26.7% lesion	21.7% mask<br>20.9% lesion	11.9% mask<br>49.3% lesion
145	ischemic	192	subcortical	0% mask<br>3.1% lesion	0.9% mask<br>45.3% lesion	0.2% mask<br>51.6% lesion
155	ischemic	732	cerebellar	0% mask<br>0% lesion	0% mask<br>0% lesion	0% mask<br>0% lesion
161	ischemic	8397	cortico-subcortical	1.2% mask<br>15% lesion	25.9% mask<br>30.6% lesion	9.2% mask<br>46.7% lesion
162	ischemic	5285	cortico-subcortical	1.8% mask<br>36% lesion	0.1% mask<br>0.1% lesion	7.6% mask<br>61.4% lesion
164	other	3650	cortical	1.9% mask<br>52.5% lesion	0% mask<br>0% lesion	3.5% mask<br>40.5% lesion
165	ischemic	255	cortical	0.2% mask<br>67.5% lesion	0% mask<br>0% lesion	0.1% mask<br>23.5% lesion
166	ischemic	2657	cortical	1.4% mask<br>55.5% lesion	0% mask<br>0% lesion	2.4% mask<br>38.9% lesion
168	ischemic	179	cortical	0.1% mask<br>41.3% lesion	0% mask<br>0% lesion	0.2% mask<br>55.9% lesion
169	ischemic	596	subcortical	0% mask<br>3.2% lesion	1.2% mask<br>20.3% lesion	1.1% mask<br>76.5% lesion
170	hemorrhagic	6182	other	0.8% mask<br>12.5% lesion	20.5% mask<br>33% lesion	6.7% mask<br>46% lesion
173	hemorrhagic	2711	subcortical	0% mask<br>0% lesion	18.2% mask<br>66.5% lesion	1% mask<br>15.3% lesion
175	ischemic	538	cerebellar	0% mask<br>0% lesion	0% mask<br>0% lesion	0% mask<br>0% lesion
178	hemorrhagic	5116	subcortical	0.7% mask<br>14.7% lesion	16.2% mask<br>31.4% lesion	6.2% mask<br>51.3% lesion
181	hemorrhagic	1804	brainstem	0% mask<br>0% lesion	0.1% mask<br>0.6% lesion	0% mask<br>0% lesion
182	hemorrhagic	660	subcortical	0% mask<br>2.7% lesion	0.1% mask<br>70.8% lesion	0% mask<br>21.4% lesion
186	ischemic	70	brainstem	0% mask<br>0% lesion	0% mask<br>0% lesion	0% mask<br>0% lesion
190	hemorrhagic	2625	cortico-subcortical	0% mask<br>50.6% lesion	0% mask<br>0.3% lesion	0% mask<br>47.5% lesion
193	ischemic	126	brainstem	0% mask<br>0% lesion	0% mask<br>0% lesion	0% mask<br>0% lesion

SI-Table 3: Individual lesion information (part 2 of 2). GM = gray matter; WM = white matter. Source data are provided as a Source Data file.

	KL divergence [ave (std)]			
	**CTRs**	**PATs (2 weeks)**	**PATs (3 months)**	**PATs (1 year)**
**Whole matrix**	0.02 (0.01)	0.02 (0.01)	0.02 (0.01)	0.02 (0.01)
**VIS**	0.51 (0.34)	0.56 (0.68)	0.56 (0.48)	0.52 (0.59)
**SMN**	1.77 (1.50)	1.82 (2.66)	2.47 (3.29)	2.08 (2.67)
**AUD**	0.52 (0.38)	0.42 (0.35)	0.49 (0.31)	0.48 (0.43)
**CON**	0.68 (1.04)	0.30 (0.30)	0.33 (0.28)	0.38 (0.41)
**VAN**	0.14 (0.09)	0.19 (0.17)	0.17 (0.13)	0.16 (0.15)
**DAN**	0.38 (0.46)	0.2 (0.26)	0.23 (0.31)	0.28 (0.29)
**FPN**	0.33 (0.37)	0.21 (0.26)	0.28 (0.40)	0.27 (0.39)
**DMN**	0.49 (0.57)	0.56 (1.01)	0.69 (1.55)	0.87 (2.19)
**None**	0.24 (0.21)	0.23 (0.15)	0.22 (0.13)	0.20 (0.13)
**SUB**	0.01 (0.01)	0.01 (0.01)	0.01 (0.01)	0.01 (0.01)

SI-Table 4: Kullback-Leibler (KL) divergence between unreduced functional connectivity (FC) and reduced FC. Source data are provided as a Source Data file.

	$\boldsymbol{f_1}$ (mean±sem)	$\boldsymbol{f_2}$ (mean±sem)	$\boldsymbol{f_3}$ (mean±sem)	$\boldsymbol{f_4}$ (mean±sem)	$\boldsymbol{f_5}$ (mean±sem)
**Control analysis** (22 subjects)	0.25 ± 0.03	0.23 ± 0.03	0.23 ± 0.02	0.17 ± 0.02	0.12 ± 0.02
**Main analysis** (40 subjects)	0.25 ± 0.03	0.22 ± 0.03	0.23 ± 0.02	0.18 ± 0.02	0.11 ± 0.01
**t-test** (p-val)	$t = 0.02$ ($p = 0.98$)	$t = 0.17$ ($p = 0.86$)	$t = -0.06$ ($p = 0.95$)	$t = -0.34$ ($p = 0.73$)	$t = 0.17$ ($p = 0.86$)

SI-Table 5: Fraction time of each Dynamical Functional State (DFS) for control subjects in the control and main analysis, together with the results of an unpaired two-sided t-test. Data are presented as mean values ± SEM. Source data are provided as a Source Data file.

	$\boldsymbol{\ell_1}$ (mean±sem)	$\boldsymbol{\ell_2}$ (mean±sem)	$\boldsymbol{\ell_3}$ (mean±sem)	$\boldsymbol{\ell_4}$ (mean±sem)	$\boldsymbol{\ell_5}$ (mean±sem)
**Control analysis** (22 subjects)	14.0 ± 1.5	12.5 ± 1.6	12.4 ± 1.0	12.0 ± 1.4	8.8 ± 1.1
**Main analysis** (40 subjects)	13.8 ± 1.4	12.2 ± 1.5	12.6 ± 1.1	12.5 ± 1.1	8.4 ± 0.9
**t-test** (p-val)	$t = 0.07$ ($p = 0.94$)	$t = 0.14$ ($p = 0.88$)	$t = -0.12$ ($p = 0.91$)	$t = -0.22$ ($p = 0.82$)	$t = 0.26$ ($p = 0.80$)

SI-Table 6: meanrage dwell time of each Dynamical Functional State (DFS) for control subjects in the control and main analysis, together with the results of an unpaired two-sided t-test. Data are presented as mean values ± SEM. Source data are provided as a Source Data file.

			to			
		**DFS1**	**DFS2**	**DFS3**	**DFS4**	**DFS5**
**DFS1**	**control analysis**		0.06 ± 0.007	0.08 ± 0.014	0.05 ± 0.009	0.04 ± 0.008
	**main analysis**		0.06 ± 0.007	0.07 ± 0.013	0.05 ± 0.009	0.04 ± 0.007
	**t-val** (p-val)		0 (1)	0.047 (0.86)	-0.12 (0.90)	-0.12 (0.90)
**DFS2**	**control analysis**	0.05 ± 0.009		0.06 ± 0.011	0.05 ± 0.009	0.04 ± 0.010
	**main analysis**	0.05 ± 0.008		0.06 ± 0.009	0.05 ± 0.009	0.05 ± 0.009
	**t-val** (p-val)	0.14 (0.89)		0.13 (0.90)	0.05 (0.96)	-0.12 (0.90)
from **DFS3**	**control analysis**	0.08 ± 0.015	0.06 ± 0.010		0.05 ± 0.009	0.04 ± 0.012
	**main analysis**	0.07 ± 0.012	0.06 ± 0.009		0.06 ± 0.010	0.03 ± 0.010
	**t-val** (p-val)	0.24 (0.81)	0.25 (0.81)		0.27 (0.79)	0.03 (0.97)
**DFS4**	**control analysis**	0.04 ± 0.010	0.05 ± 0.010	0.06 ± 0.009		0.03 ± 0.007
	**main analysis**	0.05 ± 0.009	0.05 ± 0.009	0.06 ± 0.009		0.03 ± 0.007
	**t-val** (p-val)	-0.3 (0.77)	0.07 (0.95)	-0.02 (0.99)		-0.3 (0.77)
**DFS5**	**control analysis**	0.04 ± 0.008	0.04 ± 0.009	0.04 ± 0.010	0.03 ± 0.005	
	**main analysis**	0.04 ± 0.007	0.04 ± 0.009	0.04 ± 0.008	0.03 ± 0.006	
	**t-val** (p-val)	0.08 (0.94)	-0.02 (0.99)	-0.02 (0.99)	-0.35 (0.73)	

SI-Table 7: Transition probability betweenDynamical Functional States (DFSs) for control subjects in the control and main analysis, together with the results of an unpaired two-sided t-test. Data are presented as mean values $\pm$ SEM. Source data are provided as a Source Data file.

Supplementary Figures

Lesion frequency map of the 47 patients considered for the analyses

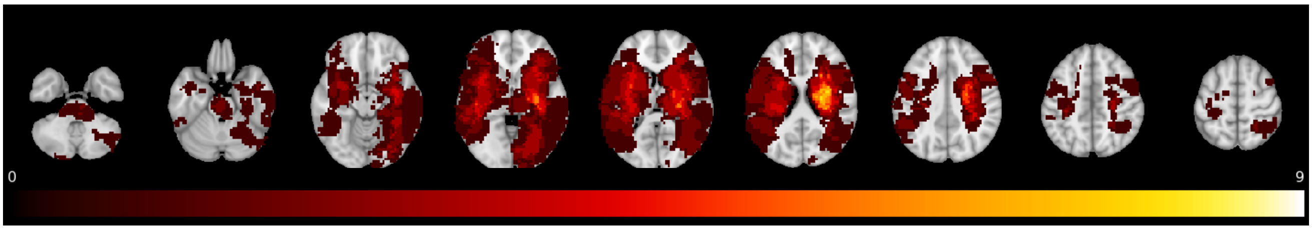

SI-Figure 1: Lesion frequancy map related to the 47 patients considered throughout the analysis

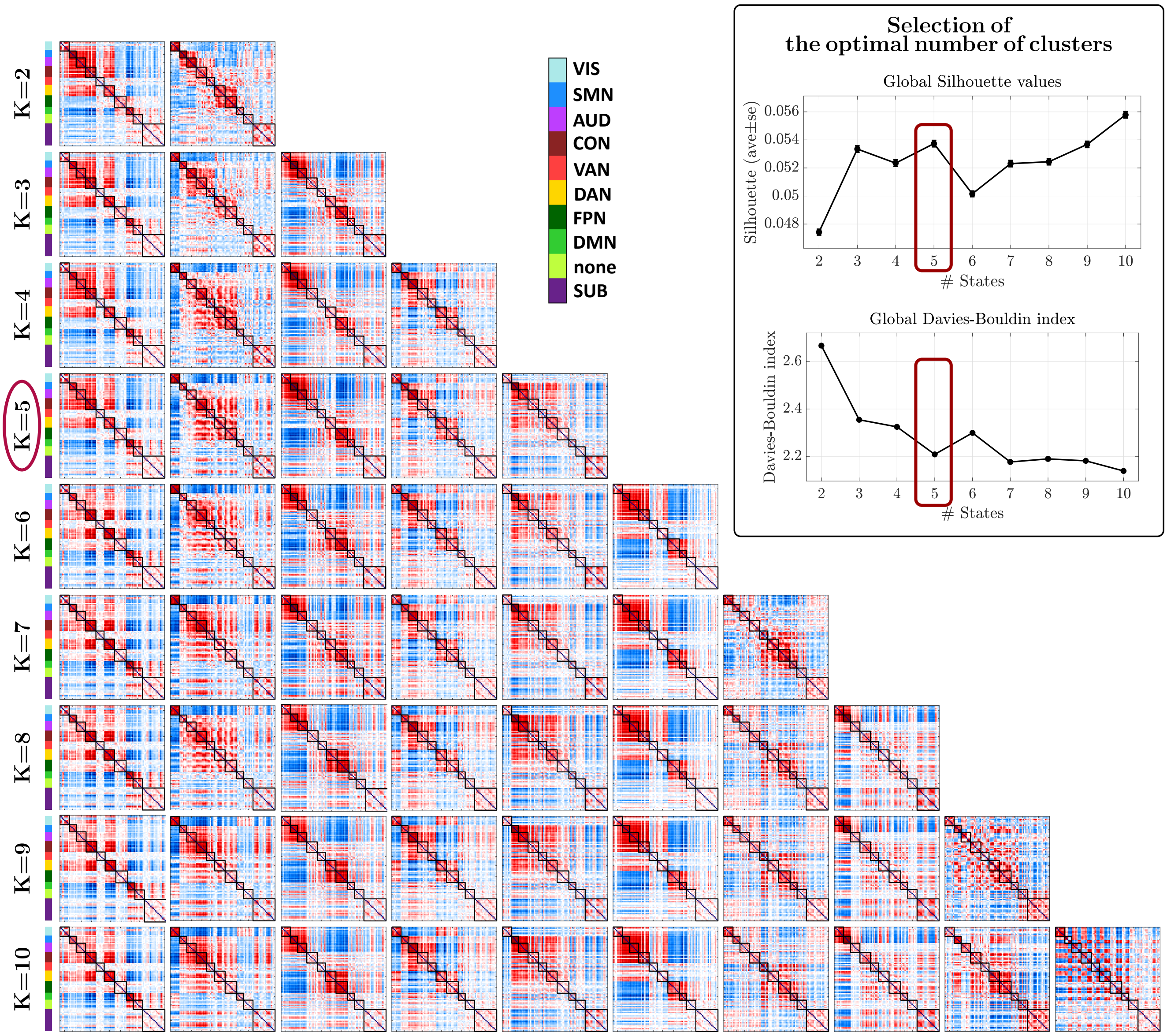

SI-Figure 2: **Dynamical Functional States for K = 2, . . . , 10.** DFSs obtained for different values of K, from 2 to 10. Through Silhouette and Davies-Bouldin indexes (in the box), in the main text we analyzed the case with K = 5. (As for the global Silhouette values, data are presented as mean values ± SEM.) Source data are provided as a Source Data file.

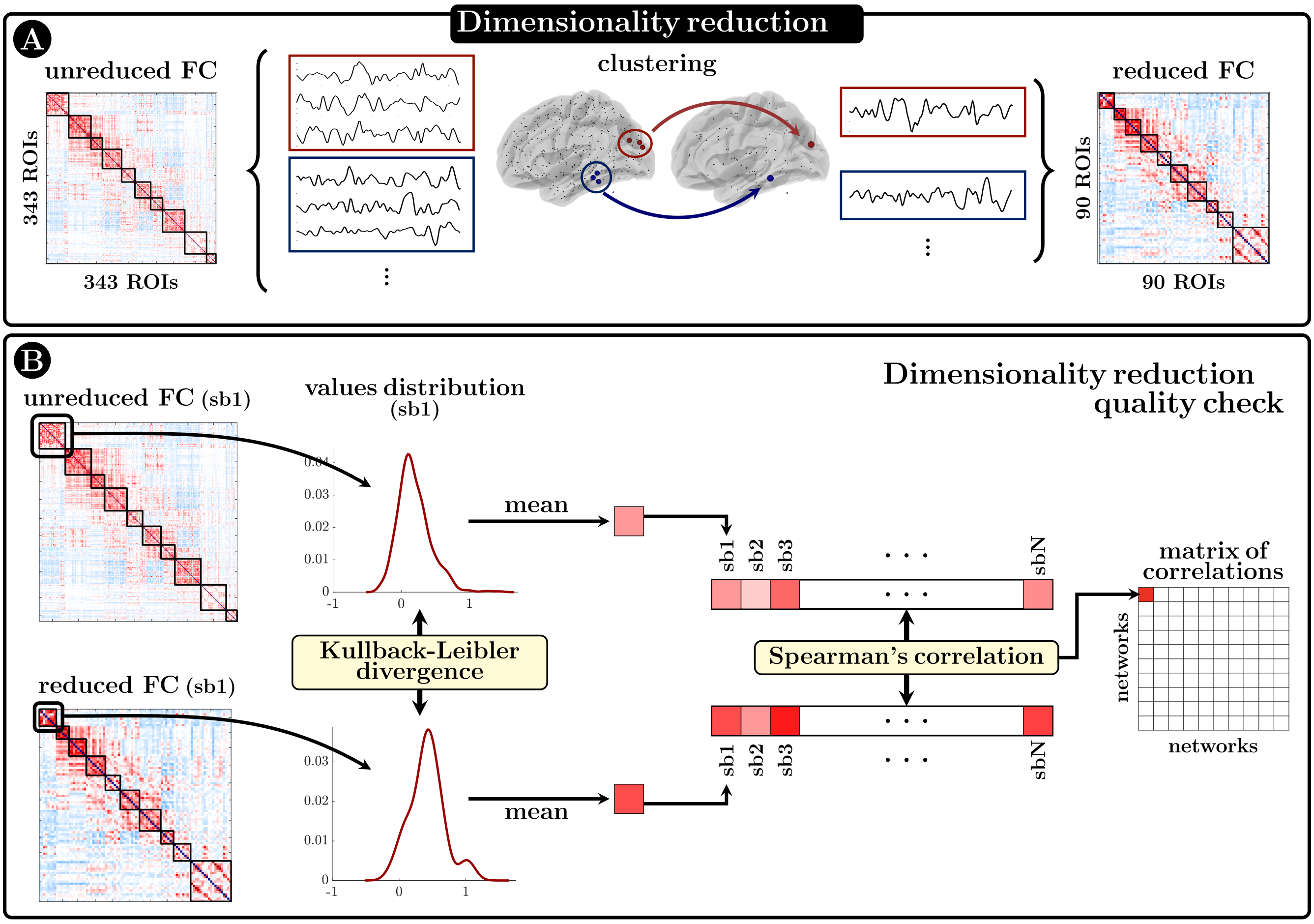

SI-Figure 3: **Methods (dimensionality reduction): A)** representation of the spatial clustering used to reduce the number of region of interests (ROIs) from 343 to 90. **B)** description of the quality check for the reduced functional connectivity (FC). The similarity between reduced and unreduced FC was evaluated by means of Kullback-Leibler divergence between the distribution of connectivity values in each within and between network, for each subject separately. To verify that the relationship among subjects remained invariant, the averaged connectivity was evaluated for each within or between network for each subject and used as input for the Spearman's correlation among the subjects' averaged connectivity for the reduced and the unreduced FC. The averaged connectivity for the reduced FC of every subjects was used also to test the significant difference between within- and between-networks connectivity (not shown).

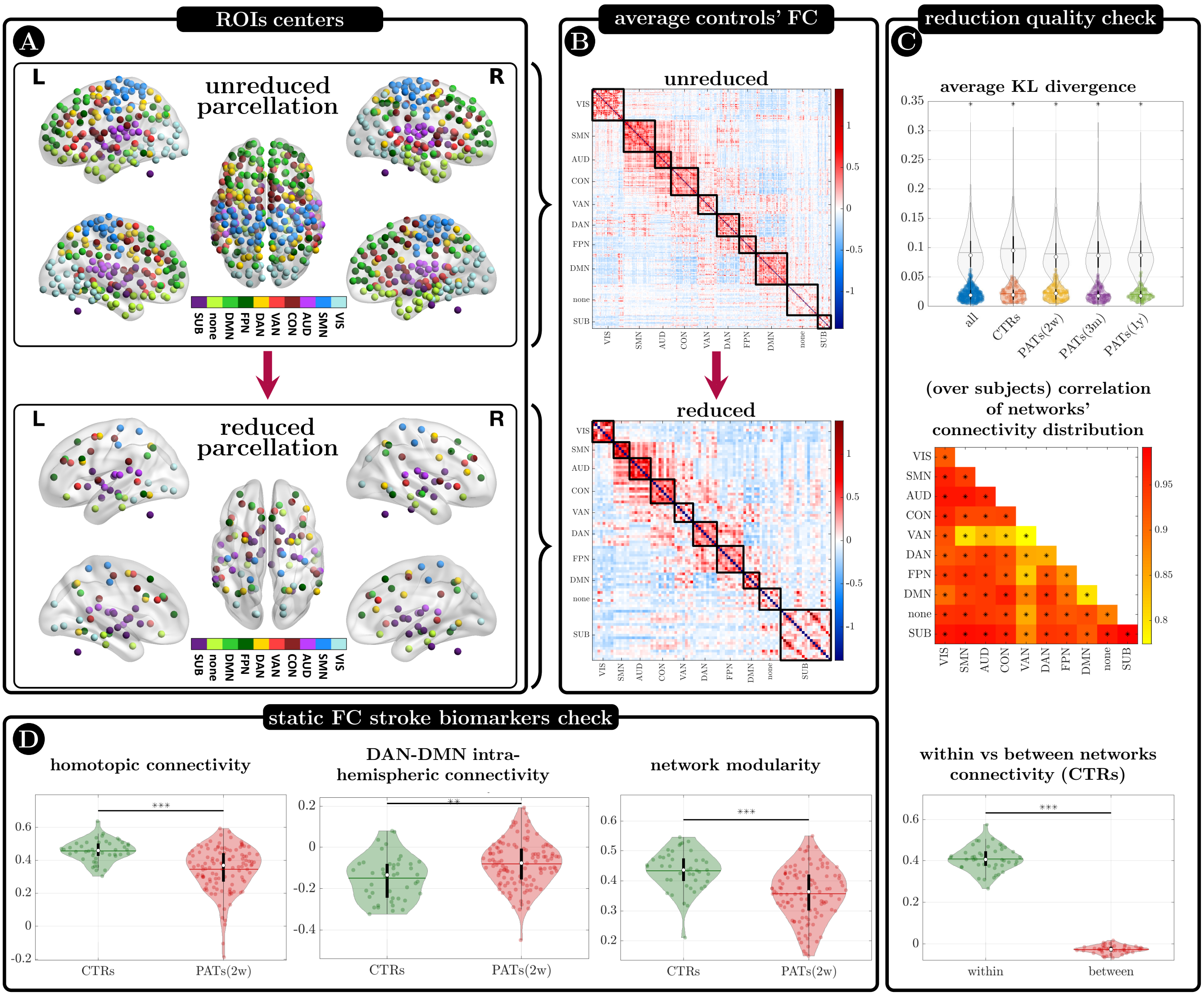

SI-Figure 4: **Dimensionality reduction quality check A)** Location of the ROIs centers of the unreduced (top) and reduced (bottom) parcellation, obtained as the results of spatial hierarchical clustering. Networks belongings are color coded. **B)** The unreduced and reduced average functional connectivity (FC) of control subjects (CTRs). **C)** Result of the quality check for the dimensionality reduction. The Kullback-Leiber divergence between ureduced and reduced FC values (top) is depicted for all subjects and for single populations in color. Each distribution is compared with the null Kullback-Leibler divergence (KLD) distribution obtained with permutations (gray), and resulted significant lower in all cases (The symbol * indicates p=0.0 for all groups) (n-CTR = 44; n-PATs(2w)=114; n-PATs(3m) = 80; n-PATs(1y) = 65; n-all = 303 samples). Moreover, the invariant relationship among subjects (n = 303) is verified by the high level of correlation obtained for each within and between network correlation values distribution (center), significantly larger then the results obtained through permutations. Finally, the reduced FC is characterized by significant larger within-network correlations than between-network correlation in control subjects (n = 44). *** stands for $p < 0.0001$ (t = 46.25, paired two-sided t-test) (bottom). Here the average over all networks is represented for CTRs, but all networks was tested also separately and for patients populations, too. **D)** Static FC biomarkers of stroke are verified also using the reduced parcellation: reduced homotopic connectivity (*** indicates $p < 0.0001$, t = 5.22, unpaired two-sided t-test), increased DAN-DMN intra-hemispheric connectivity ** indicates $p < 0.001$, $t = -3.55$, unpaired two-sided t-test), and decreased network modularity (*** indicates $p < 0.0001$, t = 5.34, unpaired two-sided t-test) (n-CTRs = 44; n-PATs(2w) = 114). In all violin plots, the white circle indicates the median, the lower and extremes of the thicker vertical line indicate the 25th and the 75th percentiles, respectively, the thiner vertical lines covers the most extreme data points, not considered outliers, and the colored horizontal line indicates the mean. Please, refer to [29] for additional details. Source data are provided as a Source Data file.

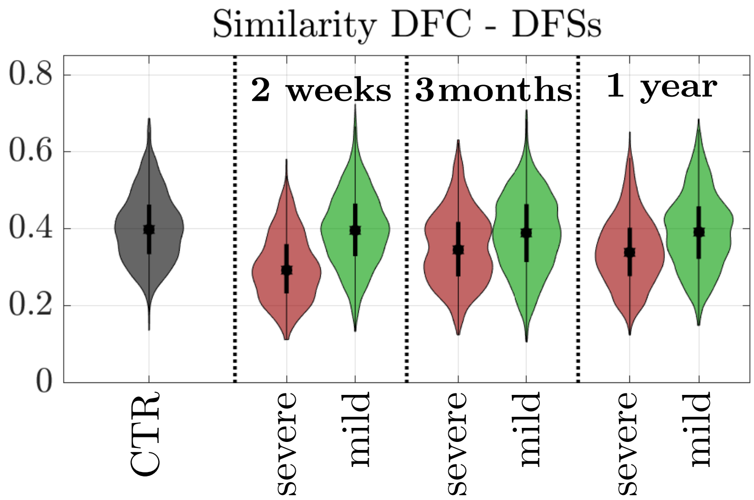

SI-Figure 5: **Analysis of similarity between the Dynamical Functional Connectivity matrix DFC evaluated at each sliding window and the Dynamical Functional State (DFS) assigned to it.** Distribution of correlation values between the Dynamical Functional Connectivity matrix DFC evaluated at sliding window and the DFS assigned to it of all sliding windows, divided for controls and patients at different time points and for different impairment severity (severe or mild; refer to SI-Section S8). (n = 270 sliding windows for eaach subject: n-CTR = 40; n-PAT (2 weeks) severe = 18; n-PAT (2 weeks) mild = 29; n-PAT (3 months) severe = 18; n-PAT (3 months) mild = 29; n-PAT (1 year) severe = 18; n-PAT (1 year) mild = 29). In all violin plots, the black central square indicates the median, the lower and extremes of the thicker vertical line indicate the 25th and the 75th percentiles, respectively, and the thiner vertical lines covers the most extreme data points, not considered outliers. Please, refer to [29] for additional details. Source data are provided as a Source Data file.

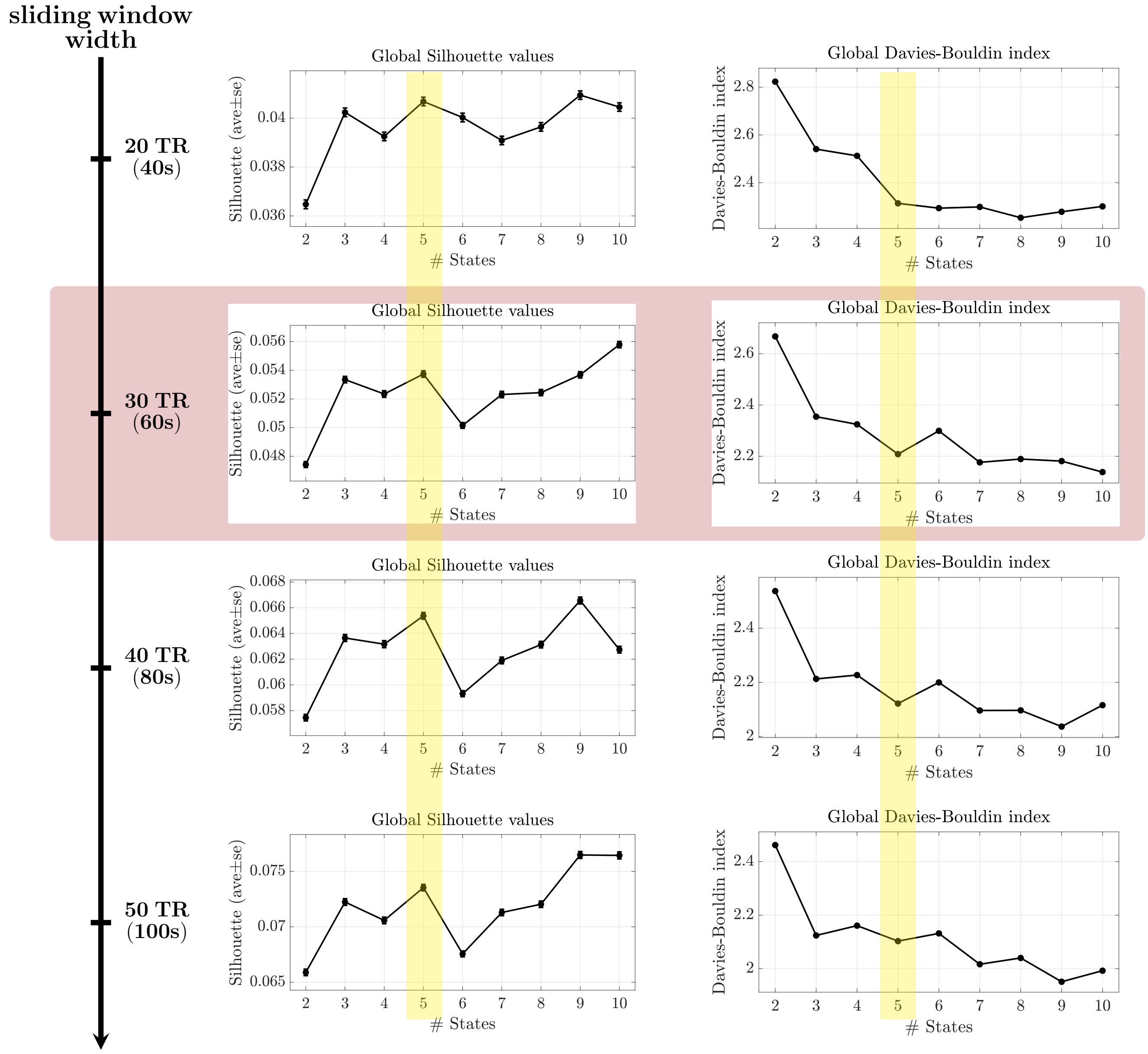

SI-Figure 6: **Impact of sliding windows width in determining the number of Dynamical Functional States (DFSs.)** As for the global Silhouette values, data are presented as mean values ± SEM. Source data are provided as a Source Data file.

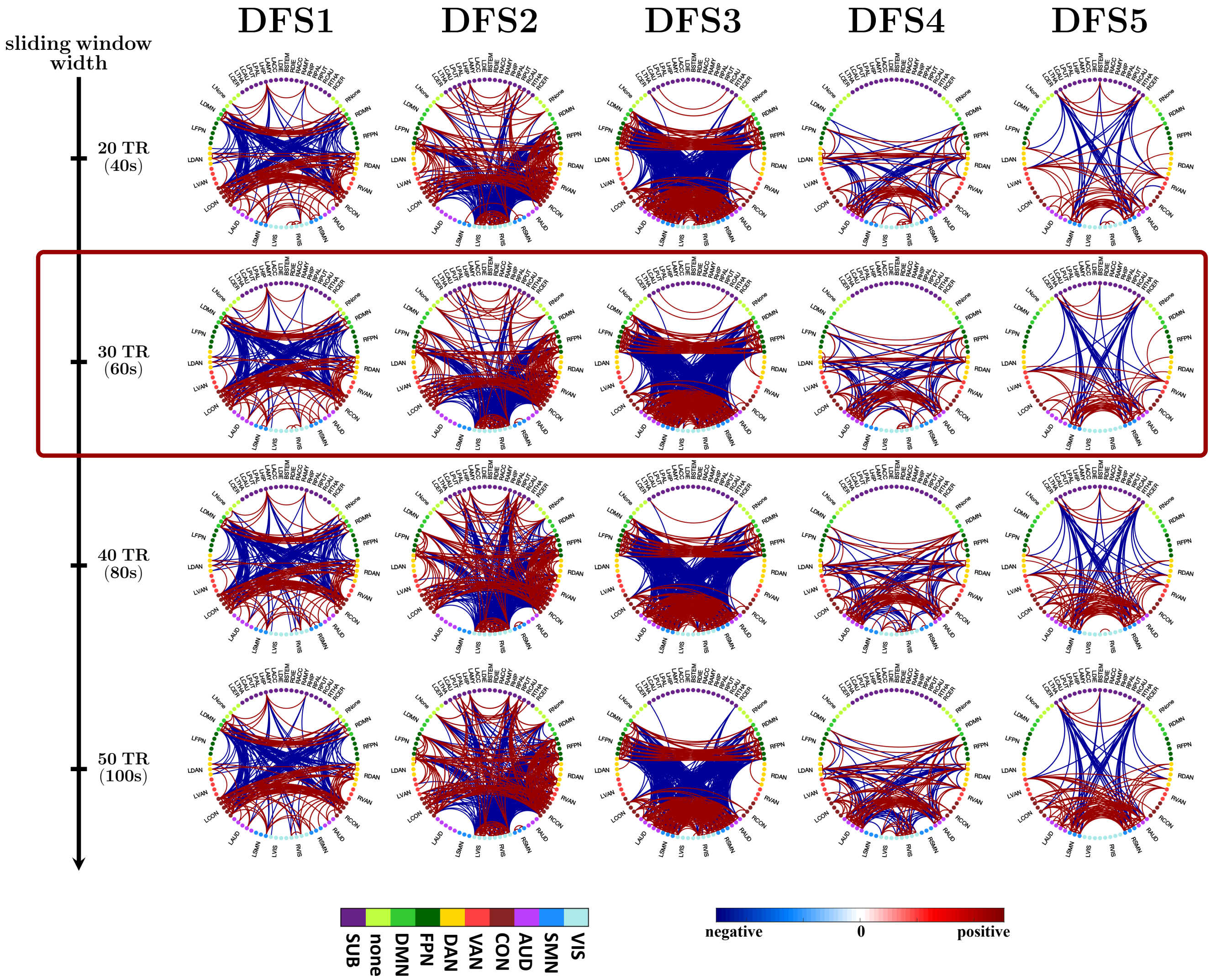

SI-Figure 7: **Impact of sliding windows width in determining the shape of each Dynamical Functional State (DFS) (graph representation).** Source data are provided as a Source Data file.

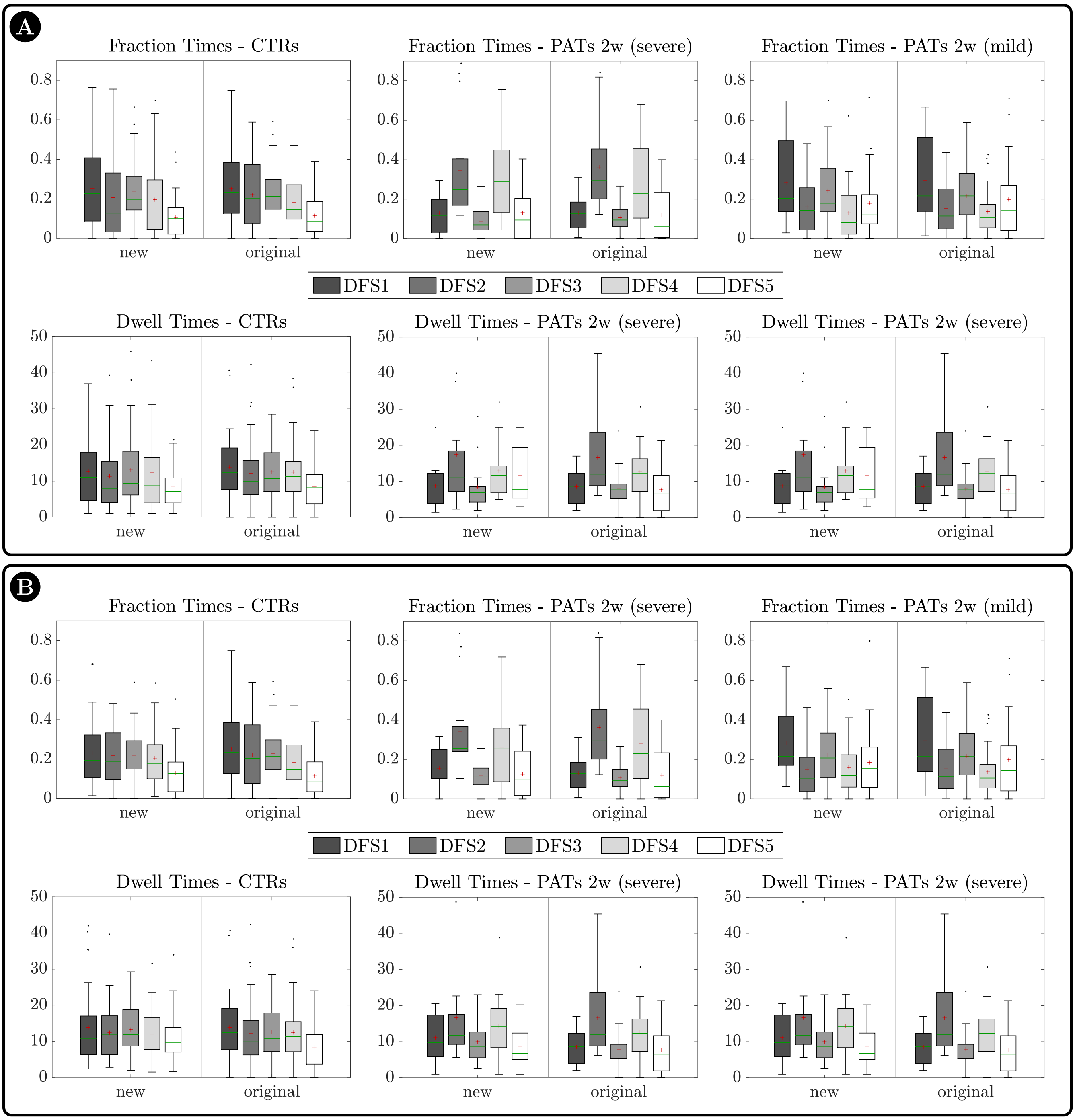

SI-Figure 8: **Fraction times and dwell times obtained with two different censoring thresholds:** a more stringent threshold of 0.25 **(A)**, and a more liberal threshold of 0.75 **(B)** (In all panels: n-CTRs = 40; n-PATs 2w (severe) = 18; n-PATs 2w (mild) = 29 for each Dynamical Functional State (DFS)). On each box: the central green line indicates the median, the red cross indicates the mean, and the bottom and top edges of the box indicate te 25th and 75th percentiles, respectively. The whiskers extend to the most extreme data points, not considered outliers (plotted individually using a dot). Source data are provided as a Source Data file.

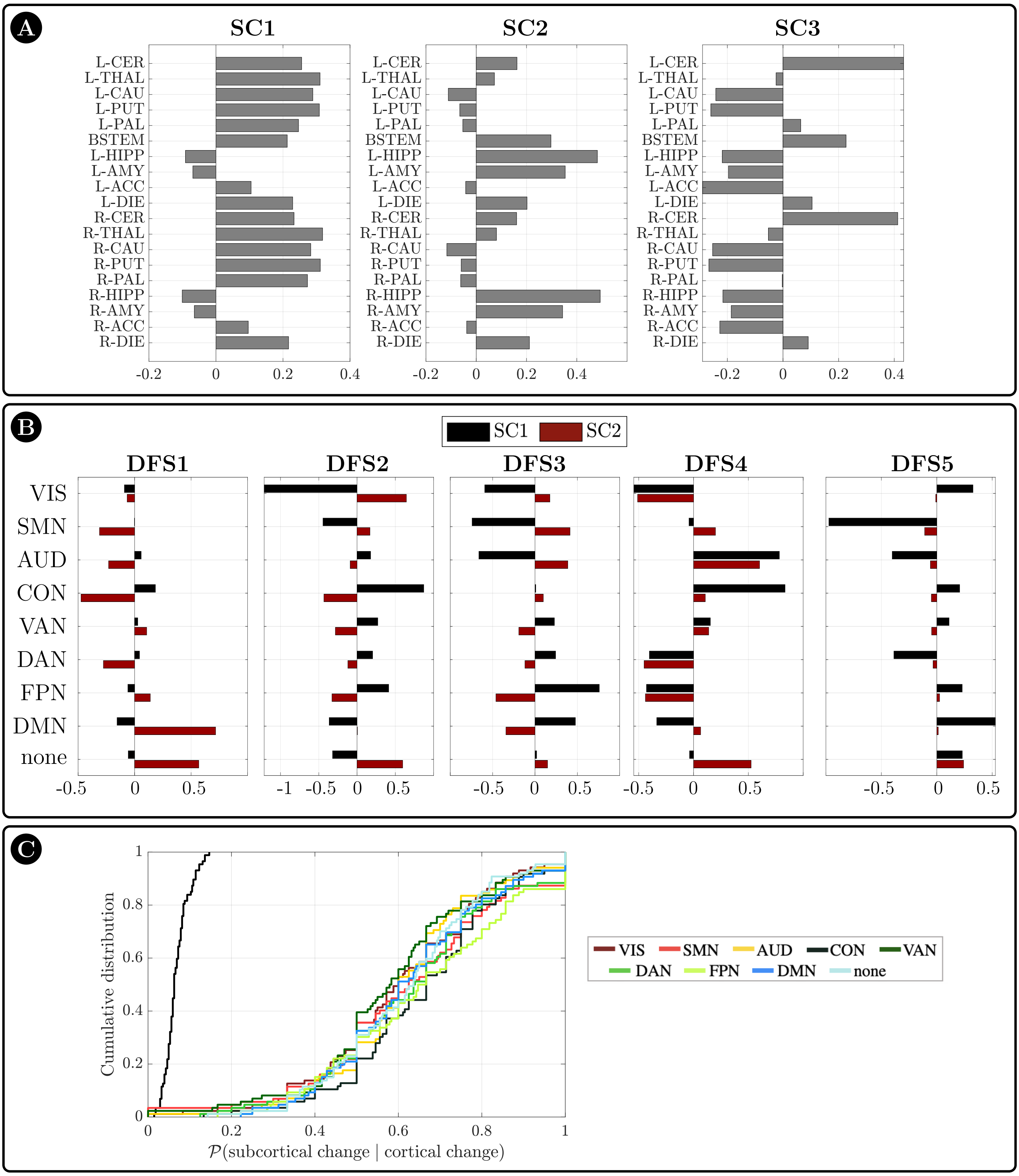

SI-Figure 9: **Results of cortical vs. subcortical reorganization pattern obtained with a more stringent censoring threshold** (0.25). **(a)** projection of the first three principal components (SCs) of subcortical functional connectivity dynamics on each subcortical region. **(b)** Connectivity between cortical networks and the first two SCs of subcortical functional connectivity dynamics. **(c)** Cumulative density function of the conditioned probability of subcortical connectivity reorganization, given a cortical connectivity reorganization. Each colored line relates to a different cortical network. The black line shows the cumulative density function under the null hypothesis of independence between cortical and subcortical changes. Source data are provided as a Source Data file. (VIS: visual network, SMN: sensory motor network, AUD: auditory network, CON: control network, VAN: ventral attention network, DAN: dorsal attention network, FPN: fronto parietal network, DMN: default mode network).

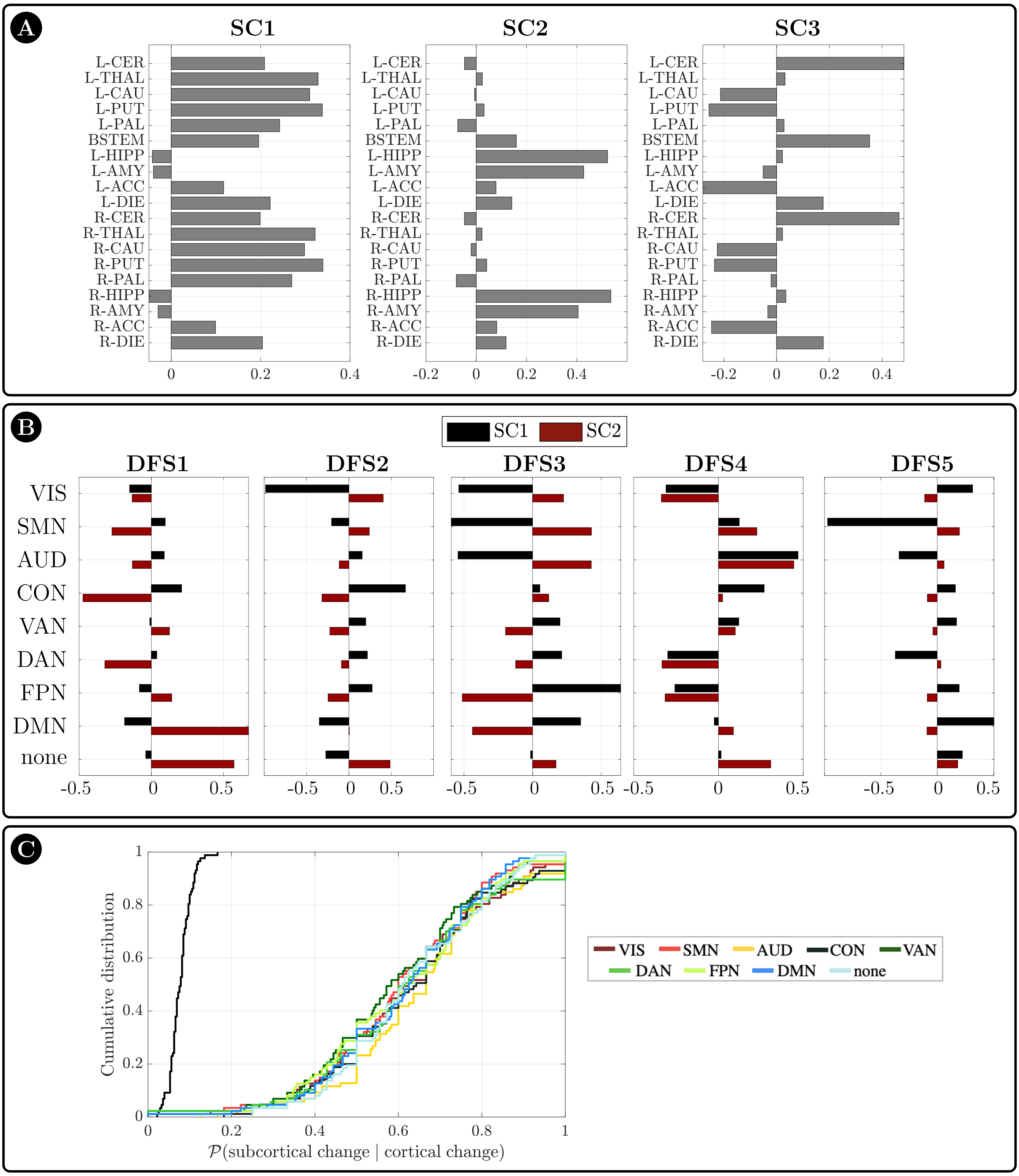

SI-Figure 10: **Results of cortical vs. subcortical reorganization pattern obtained with a more liberal censoring threshold** (0.75). **(a)** projection of the first three principal components (SCs) of subcortical functional connectivity dynamics on each subcortical region. **(b)** Connectivity between cortical networks and the first two SCs of subcortical functional connectivity dynamics. **(c)** Cumulative density function of the conditioned probability of subcortical connectivity reorganization, given a cortical connectivity reorganization. Each colored line relates to a different cortical network. The black line shows the cumulative density function under the null hypothesis of independence between cortical and subcortical changes. Source data are provided as a Source Data file. (VIS: visual network, SMN: sensory motor network, AUD: auditory network, CON: control network, VAN: ventral attention network, DAN: dorsal attention network, FPN: fronto parietal network, DMN: default mode network).

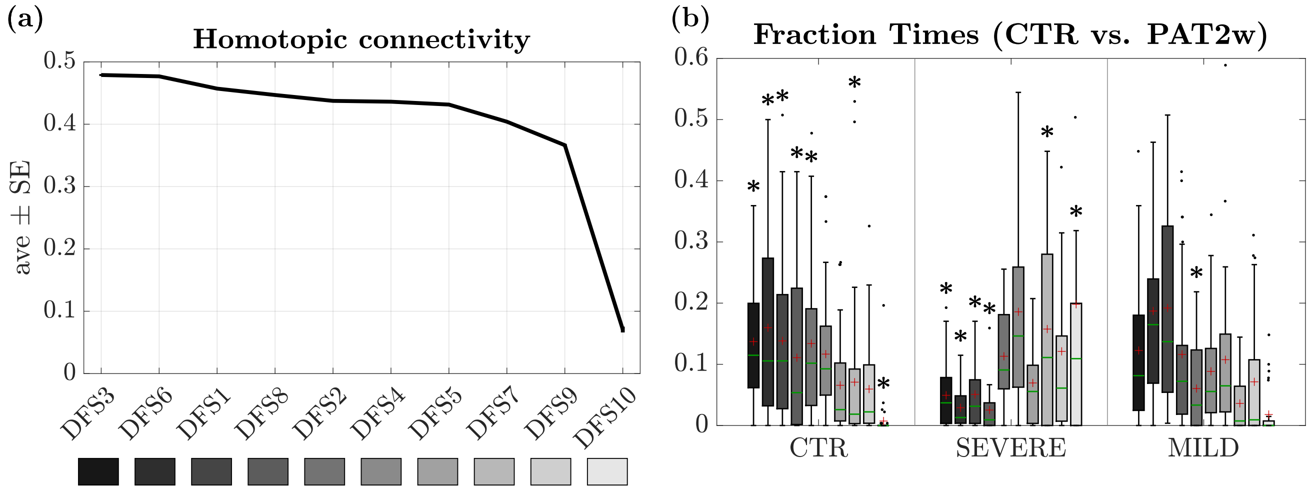

SI-Figure 11: **Homotopic connectivity and fraction times with $\mathbf{K = 10}$. (a)** Total homotopic functional connectivity (FC) for Dynamical Functional States (DFSs) obtained with $K = 10$ **(b)** Fraction times for DFSs obtained with $K = 10$ (sorted according to descending homotopic FC) (n-CTRs = 40; n-PATs 2w (severe) = 18; n-PATs 2w (mild) = 29 for each DFS). The significance between each pair of groups has been tested independently for each of the 10 DFSs through two-sided non-parametric permutation tests, and FDR correction for 30 comparisons. The symbol * indicates $p < 0.05$ after FDR correction. On each box: the central green line indicates the median, the red cross indicates the mean, and the bottom and top edges of the box indicate te 25th and 75th percentiles, respectively. The whiskers extend to the most extreme data points, not considered outliers (plotted individually using a dot). Source data are provided as a Source Data file.

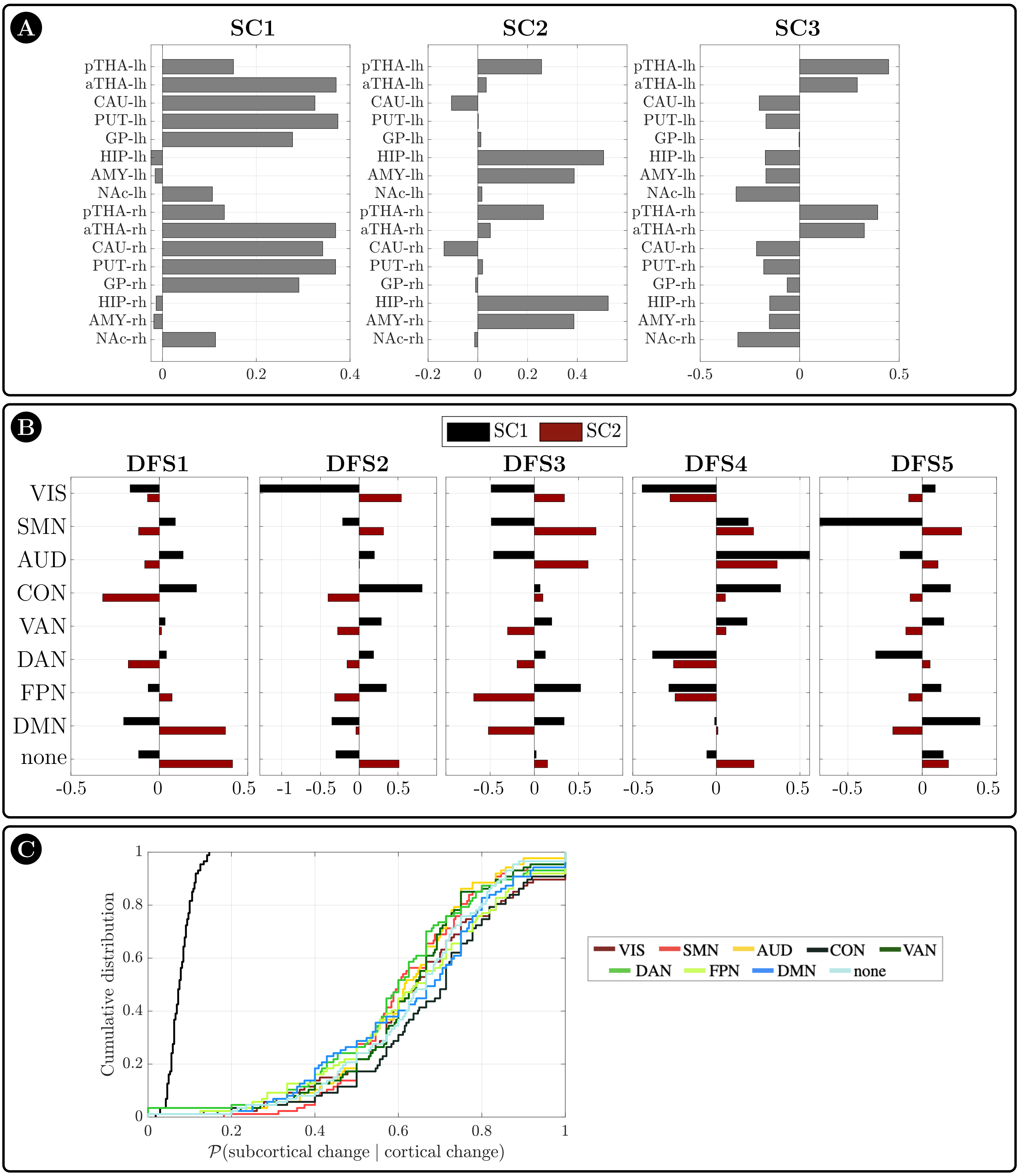

SI-Figure 12: **Results of cortical vs. subcortical reorganization pattern obtained with the subcortical parcellation by Tian et al.** **(a)** projection of the first three PCs of subcortical FC dynamics on each subcortical region, using the 16-region subcortical parcellation by Tian et al. **(b)** Connectivity between cortical networks and the first two principal components (SCs) of subcortical functional connectivity dynamics, using the 16-region parcellation by Tian et al[2]. **(c)** Cumulative density function of the conditioned probability of subcortical connectivity reorganization, given a cortical connectivity reorganization, using the 16-region subcortical parcellation by Tian et al[2]. Each colored line relates to a different cortical network. The black line shows the cumulative density function under the null hypothesis of independence between cortical and subcortical changes. Source data are provided as a Source Data file. (VIS: visual network, SMN: sensory motor network, AUD: auditory network, CON: control network, VAN: ventral attention network, DAN: dorsal attention network, FPN: fronto parietal network, DMN: default mode network).

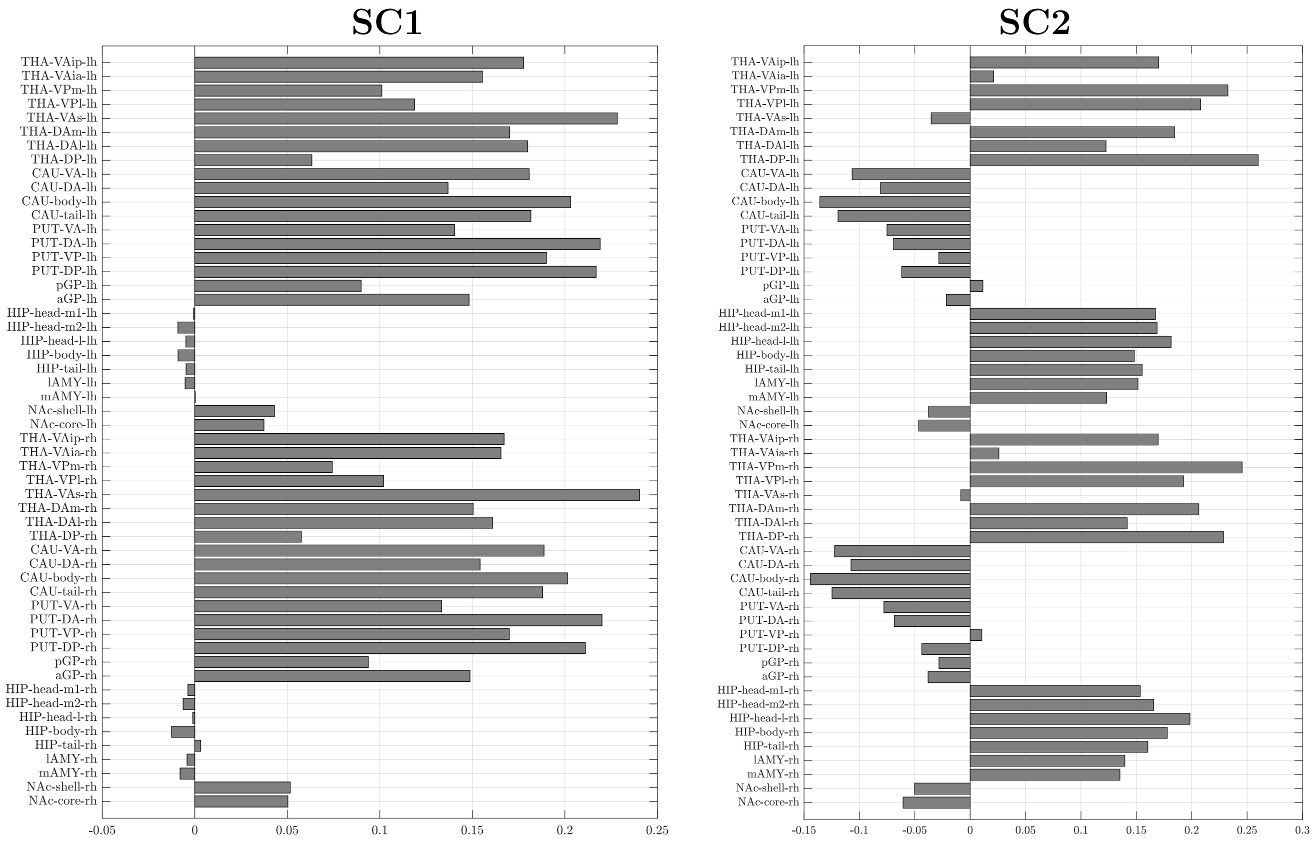

SI-Figure 13: **Projection of the first two principal components (SCs) of subcortical functional concetivity dynamics on each subcortical region, using the 54-region subcortical parcellation by Tian et al[2]**. Source data are provided as a Source Data file.

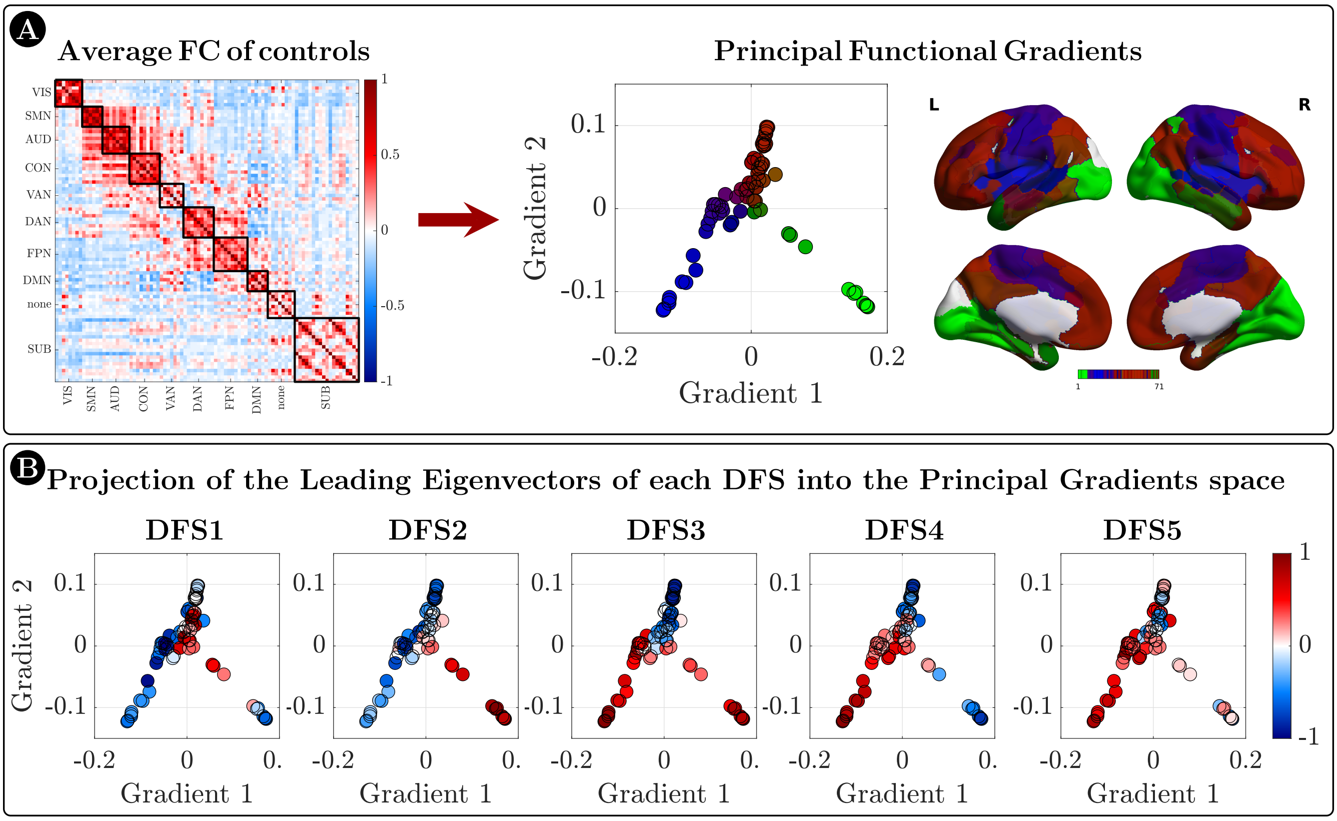

SI-Figure 14: **Representation of the Principal Functional Gradients and the DFSs projection onto them.** **A)** Starting from the average functional connectivity (FC) evaluated among the control population (left), the first two principal functional gradients have been evaluated as in Margulies et al.. Each dot represents a cortical ROI (as displayed in the brain surface on the right), projected onto the space of the first two gradients. **B)** For each DFS, the values of each ROI in the related leading eigenvector has been used as color-map for the projected points onto the gradients space. Source data are provided as a Source Data file.

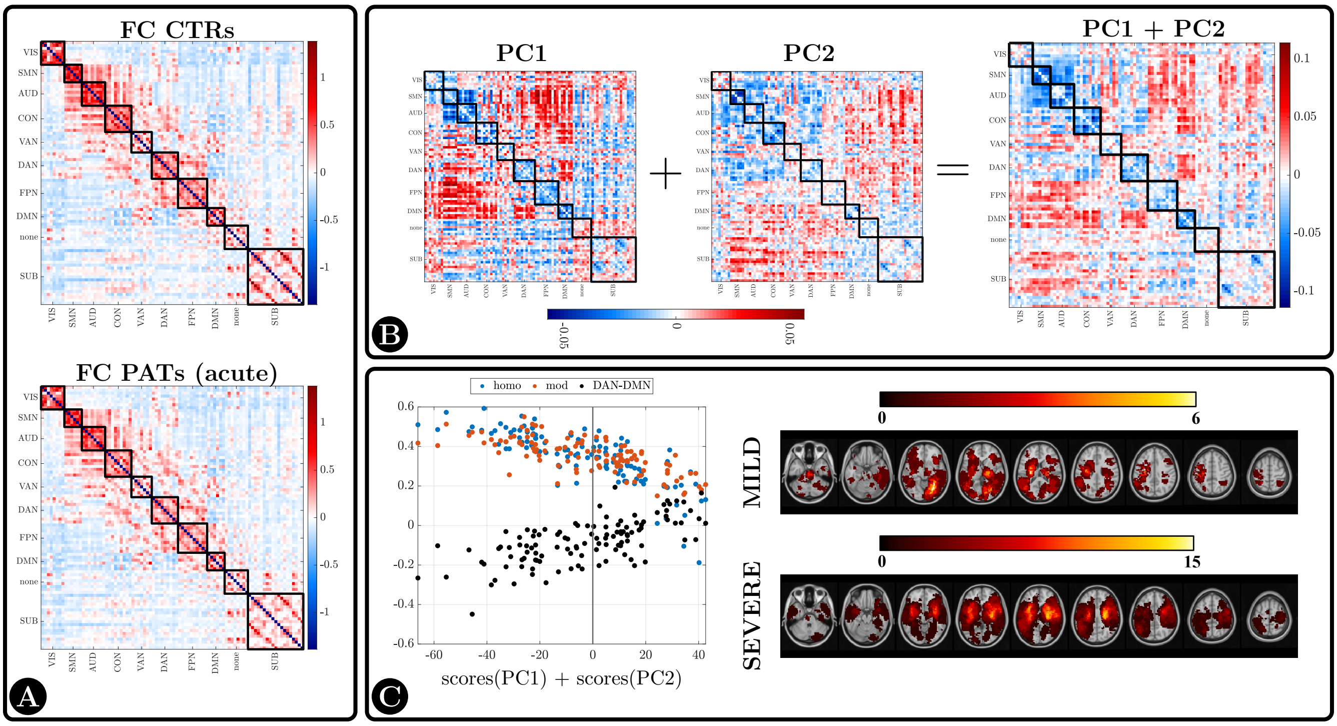

SI-Figure 15: **Results of the static principal component analysis. A)** Average functional connectivity (FC) of healthy subjects (CTRs) and sub-acute patients (PATs) (including patients not selected for the dynamical analysis). **B)** Definition of the spatial FC component used to characterize the static FC impairments of patients. Specifically, a PCA on the patients' FC (z-scored w.r.t. average CTRs FC) was conduced among patients not selected for dynamic analyses. The sum of the first two principal components (PCs) (the only ones that respected the selection rules) was used for a compact representation of stroke static impairments. **C)** After projecting all patients FC onto the static PCs space, the relationship between the sum of the scores of static principal components 1 and 2 values and static stroke biomarkers was tested. A scatter plot of this sum vs homotopic connectivity (homo, blue), network modularity (mod, red) and DAN-DMN intra-hemispheric connectivity (black) is depicted on the left. Finally, patients was grouped based on the sign of the sum of the scores of static principal components 1 and 2 (positive sum as severe patients, and negative sum as mild patients), and the frequency map of lesions for the two groups is represented on the right. Patients overlapping is color-coded. Source data are provided as a Source Data file.

Principal Components Analysis of Dynamical Measures

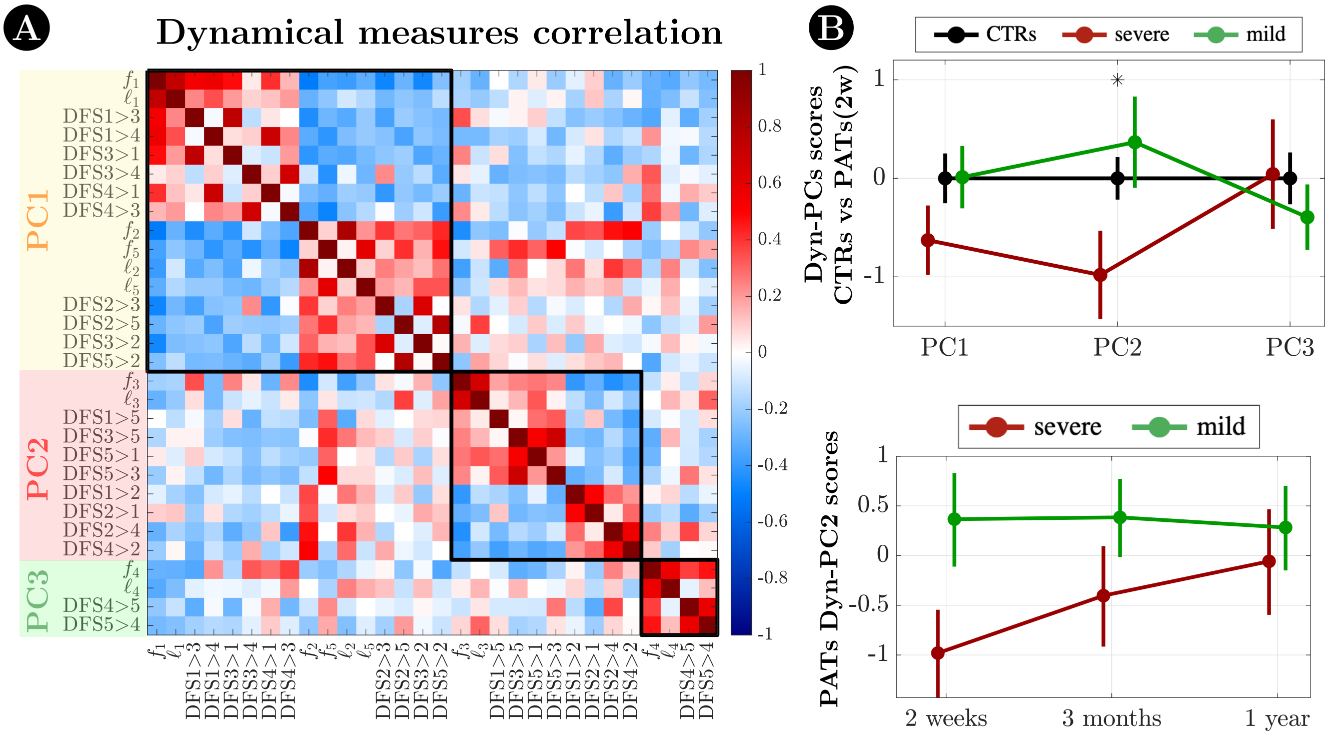

SI-Figure 16: **PCA on dynamical measures**. **A)** Matrix of correlation among all the dynamical measures of healthy subjects (CTRs), sorted w.r.t. their dynamical principal components (Dyn-PCs) loadings. **B)**: mean (± confidence interval) Dyn-PCs scores for CTRs and acute patients. A (repeated measures) ANOVA showed a significant interaction between Dyn-PCs and condition ($p<0.001$), with lower Dyn-PC2 scores for severe patients ($n = 18$) than CTRs ($n = 40$) ($p=0.004$, $t=3.93$, unpaired two-sided t-test, Bonferroni corrected) and mild patients ($n = 29$) ($p<0.001$, $t=-5.11$, unpaired two-sided t-test, Bonferroni corrected). A comparsion (repeated measures ANOVA) between the evolution of each Dyn-PC from 2 weeks to 1 year, showed a significant interaction with the level of patients' abnormalities (severe or mild), as regards Dyn-PC2 ($p = 0.039$). Indeed, the significant difference observed at 2 weeks between groups, did not hold anymore at 3 and 12 months. (For each time point: n-severe = 18 and n-mild = 29). Source data are provided as a Source Data file.

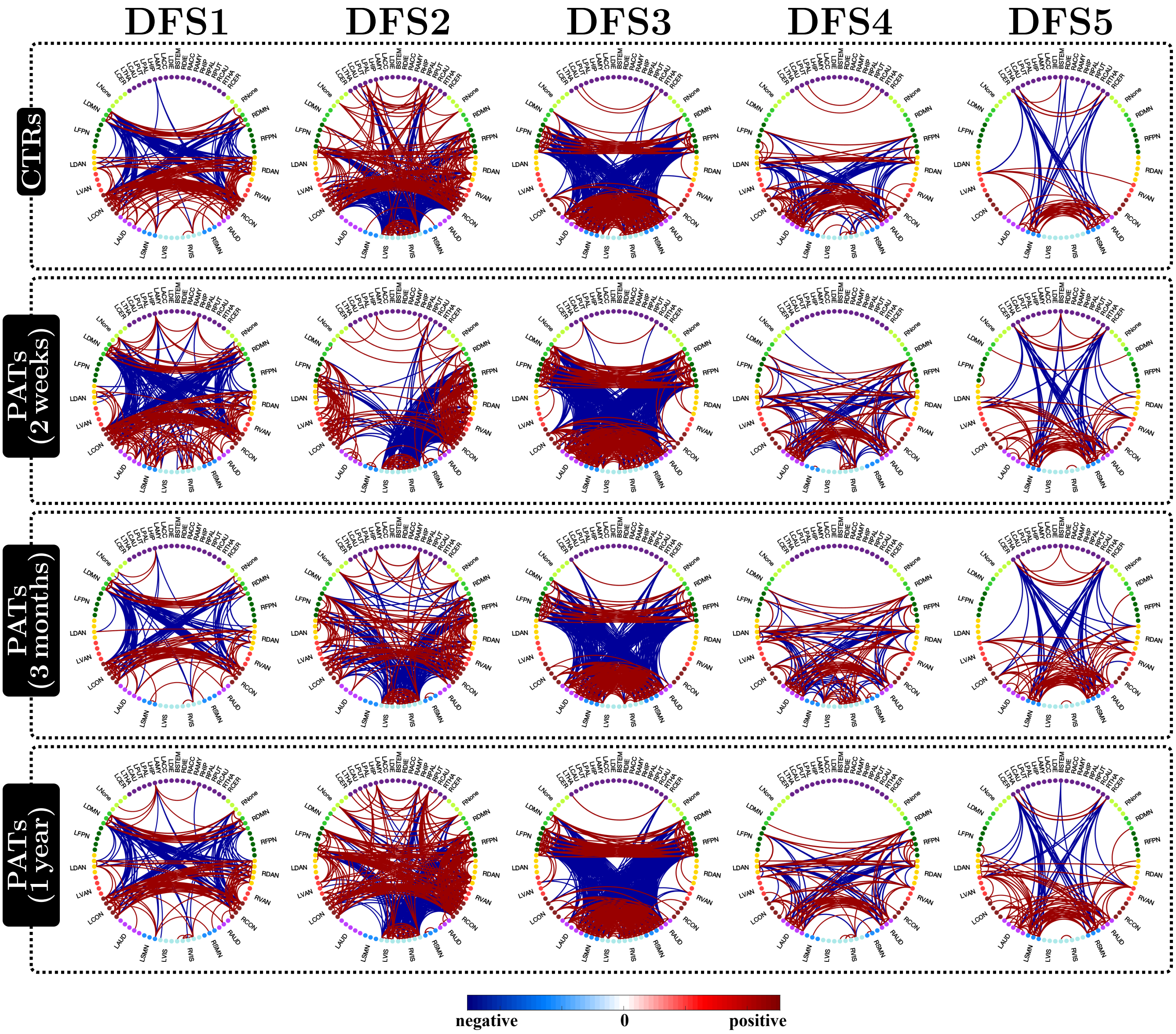

SI-Figure 17: **Dynamical Functional States for each group.** Given the assignment of all sliding window to a specif Dynamical Functional State (DFS), though the K-means clustering on all subjects and population concatenated, for each group of patients, the sliding windows assigned to each DFS were used to estimate the population-specific representation of the DFS. Interestingly, DFS2 differs between healthy subjects (CTRs) and patients (PATs), especially at acute stage. Specifically, sub-acute patients (PATs (2 weeks)) show less inter-hemispheric within-network connectivity, and reduced VIS (visual network) anticorrelation in the left hemisphere. After 3 and 12 months, DFS2 returns to be more similar to CTRs. Source data are provided as a Source Data file.

References

1. Gordon, E. M. *et al.* Generation and Evaluation of a Cortical Area Parcellation from Resting-State Correlations. *Cerebral Cortex* **26**, 288–303 (2016). URL https://academic.oup.com/cercor/article-lookup/doi/10.1093/cercor/bhu239.

2. Tian, Y., Margulies, D. S., Breakspear, M. & Zalesky, A. Topographic organization of the human subcortex unveiled with functional connectivity gradients. *Nature Neuroscience* **23**, 1421–1432 (2020). URL https://doi.org/10.1038/s41593-020-00711-6.

3. Siegel, J. S. *et al.* Disruptions of network connectivity predict impairment in multiple behavioral domains after stroke. *Proceedings of the National Academy of Sciences* **113**, E4367–E4376 (2016). URL http://www.pnas.org/lookup/doi/10.1073/pnas.1521083113.

4. Ramsey, L. E. *et al.* Normalization of network connectivity in hemispatial neglect recovery. *Annals of Neurology* **80**, 127–141 (2016). URL http://doi.wiley.com/10.1002/ana.24690.

5. Corbetta, M. *et al.* Common Behavioral Clusters and Subcortical Anatomy in Stroke. *Neuron* **85**, 927–941 (2015). URL http://dx.doi.org/10.1016/j.neuron.2015.02.027https://linkinghub.elsevier.com/retrieve/pii/S0896627315001427.15334406.

6. Siegel, J. S. *et al.* Re-emergence of modular brain networks in stroke recovery. *Cortex* **101**, 44–59 (2018). URL https://doi.org/10.1016/j.cortex.2017.12.019https://linkinghub.elsevier.com/retrieve/pii/S0010945217304276.

7. Griffis, J. C., Metcalf, N. V., Corbetta, M. & Shulman, G. L. Structural Disconnections Explain Brain Network Dysfunction after Stroke. *Cell Reports* **28**, 2527–2540.e9 (2019). URL https://doi.org/10.1016/j.celrep.2019.07.100https://linkinghub.elsevier.com/retrieve/pii/S2211124719310162.

8. Griffis, J. C., Metcalf, N. V., Corbetta, M. & Shulman, G. L. Damage to the shortest structural paths between brain regions is associated with disruptions of resting-state functional connectivity after stroke. *NeuroImage* **210**, 116589 (2020). URL https://linkinghub.elsevier.com/retrieve/pii/S1053811920300768.

9. Ward, J. H. Hierarchical Grouping to Optimize an Objective Function. *Journal of the American Statistical Association* **58**, 236–244 (1963). URL

https://www.tandfonline.com/doi/abs/10.1080/01621459.1963.10500845http://www.tandfonline.com/doi/abs/10.1080/01621459.1963.10500845.

10. Rousseeuw, P. J. Silhouettes: a graphical aid to the interpretation and validation of cluster analysis. *Journal of Computational and Applied Mathematics* **20**, 53–65 (1987). URL https://linkinghub.elsevier.com/retrieve/pii/0377042787901257.

11. Cover, T. M. & Thomas, J. A. *Elements of Information Theory* (1991).

12. He, B. J. *et al.* Breakdown of Functional Connectivity in Frontoparietal Networks Underlies Behavioral Deficits in Spatial Neglect. *Neuron* **53**, 905–918 (2007). URL https://linkinghub.elsevier.com/retrieve/pii/S0896627307001122.

13. Tang, C. *et al.* Decreased functional connectivity of homotopic brain regions in chronic stroke patients: a resting state fMRI study. *PLOS ONE* **11**, e0152875 (2016). URL https://dx.plos.org/10.1371/journal.pone.0152875.

14. Carter, A. R. *et al.* Resting state inter-hemispheric fMRI connectivity predicts performance after stroke. *Annals of Neurology* 365—-375 (2010). URL http://doi.wiley.com/10.1002/ana.21905.

15. Park, C.-h. *et al.* Longitudinal changes of resting-state functional connectivity during motor recovery after stroke. *Stroke* **42**, 1357–1362 (2011). URL https://www.ahajournals.org/doi/10.1161/STROKEAHA.110.596155.

16. Wu, W. *et al.* Impaired neuronal synchrony after focal ischemic stroke in elderly patients. *Clinical Neurophysiology* **122**, 21–26 (2011). URL https://linkinghub.elsevier.com/retrieve/pii/S1388245710005006.

17. Golestani, A.-M., Tymchuk, S., Demchuk, A. & Goodyear, B. G. Longitudinal Evaluation of Resting-State fMRI After Acute Stroke With Hemiparesis. *Neurorehabilitation and Neural Repair* **27**, 153–163 (2013). URL http://journals.sagepub.com/doi/10.1177/1545968312457827.

18. New, A. B. *et al.* Altered resting-state network connectivity in stroke patients with and without apraxia of speech. *NeuroImage: Clinical* **8**, 429–439 (2015). URL https://linkinghub.elsevier.com/retrieve/pii/S2213158215000558.

19. Baldassarre, A. *et al.* Large-scale changes in network interactions as a physiological signature of spatial neglect. *Brain* **137**, 3267–3283 (2014).

URL https://academic.oup.com/brain/article-lookup/doi/10.1093/brain/awu297.

20. Eldaief, M. C., McMains, S., Hutchison, R. M., Halko, M. A. & Pascual-Leone, A. Reconfiguration of intrinsic functional coupling patterns following circumscribed network lesions. *Cerebral Cortex* bhw139 (2016). URL http://cercor.oxfordjournals.org/cgi/doi/10.1093/cercor/bhw139.

21. Gratton, C., Nomura, E. M., Pérez, F. & D'Esposito, M. Focal Brain Lesions to Critical Locations Cause Widespread Disruption of the Modular Organization of the Brain. *Journal of Cognitive Neuroscience* **24**, 1275–1285 (2012).

22. Rubinov, M. & Sporns, O. Complex network measures of brain connectivity: Uses and interpretations. *NeuroImage* **52**, 1059–1069 (2010). URL https://linkinghub.elsevier.com/retrieve/pii/S105381190901074X.

23. Leonardi, N. & Van De Ville, D. On spurious and real fluctuations of dynamic functional connectivity during rest. *NeuroImage* **104**, 430–436 (2015). URL http://www.sciencedirect.com/science/article/pii/S1053811914007496https://linkinghub.elsevier.com/retrieve/pii/S1053811914007496.

24. Hutchison, R. M. *et al.* Dynamic functional connectivity: promise, issues, and interpretations. *Neuroimage* **80**, 360–378 (2013). URL http://dx.doi.org/10.1016/j.neuroimage.2013.05.079.

25. Smith, S. M. *et al.* Temporally-independent functional modes of spontaneous brain activity. *Proceedings of the National Academy of Sciences* **109**, 3131–3136 (2012). URL http://www.pnas.org/cgi/doi/10.1073/pnas.1121329109.

26. Ortiz, G. A. & Sacco, R. L. National Institutes of Health Stroke Scale (NIHSS). In Balakrishnan, N. *et al.* (eds.) *Wiley StatsRef: Statistics Reference Online*, stat06823 (John Wiley & Sons, Ltd, Chichester, UK, 2014). URL http://doi.wiley.com/10.1002/9781118445112.stat06823.

27. Salvalaggio, A., De Filippo De Grazia, M., Zorzi, M., Thiebaut de Schotten, M. & Corbetta, M. Post-stroke deficit prediction from lesion and indirect structural and functional disconnection. *Brain* **143**, 2173–2188 (2020). URL https://academic.oup.com/brain/article/143/7/2173/5861020.

28. Ramsey, L. E. *et al.* Behavioural clusters and predictors of performance during recovery from stroke. *Nature Human Behaviour* **1**, 0038 (2017). URL http://dx.doi.org/10.1038/s41562-016-0038http://www.nature.com/articles/s41562-016-0038.

29. Hintze, J. L. & Nelson, R. D. *The American Statistician* **52**, 181–184 (1998).
