# Supplementary material for: Subcortical-cortical dynamical states of the human brain and their breakdown in stroke
Source: Nat Commun. 2022 Aug 29;13:5069. doi: 10.1038/s41467-022-32304-1 (PMC9424299; doi:10.1038/s41467-022-32304-1)
Supplement: Supplementary file 3 — Reporting Summary [file 41467_2022_32304_MOESM3_ESM.pdf]

nature portfolio

Corresponding author(s): Chiara Favaretto
Maurizio Corbetta

Last updated by author(s): Jul 11, 2022

Reporting Summary

Nature Portfolio wishes to improve the reproducibility of the work that we publish. This form provides structure for consistency and transparency in reporting. For further information on Nature Portfolio policies, see our Editorial Policies and the Editorial Policy Checklist.

Statistics

For all statistical analyses, confirm that the following items are present in the figure legend, table legend, main text, or Methods section.

n/a	Confirmed	
☐	☒	The exact sample size (*n*) for each experimental group/condition, given as a discrete number and unit of measurement
☐	☒	A statement on whether measurements were taken from distinct samples or whether the same sample was measured repeatedly
☐	☒	The statistical test(s) used AND whether they are one- or two-sided<br>*Only common tests should be described solely by name; describe more complex techniques in the Methods section.*
☐	☒	A description of all covariates tested
☐	☒	A description of any assumptions or corrections, such as tests of normality and adjustment for multiple comparisons
☐	☒	A full description of the statistical parameters including central tendency (e.g. means) or other basic estimates (e.g. regression coefficient) AND variation (e.g. standard deviation) or associated estimates of uncertainty (e.g. confidence intervals)
☐	☒	For null hypothesis testing, the test statistic (e.g. *F*, *t*, *r*) with confidence intervals, effect sizes, degrees of freedom and *P* value noted<br>*Give P values as exact values whenever suitable.*
☒	☐	For Bayesian analysis, information on the choice of priors and Markov chain Monte Carlo settings
☐	☒	For hierarchical and complex designs, identification of the appropriate level for tests and full reporting of outcomes
☐	☒	Estimates of effect sizes (e.g. Cohen's *d*, Pearson's *r*), indicating how they were calculated

Our web collection on statistics for biologists contains articles on many of the points above.

Software and code

Policy information about availability of computer code

Data collection	No software was used to collect data
Data analysis	Software used in previous works to derived some data used for further analyses:<br><br>- MATLAB 2015a (in Siegel et al., 2016, PNAS and Griffis et al., Cell Reports, 2019)<br>- DSI studio 2019 (http://dsi-studio.labsolver.org/; used in Griffis et al., Cell eports, 2019)<br>- FreeSurfer V6 (https://surfer.nmr.mgh.harvard.edu/; used in Griffis et al., Cell eports, 2019)<br>- Connectome workbench v1.5.0 (https://www.humanconnectome.org/software/get-connectome-workbench; used in Griffis et al., Cell eports, 2019)<br>- GRETNA 22.0 (https://www.nitrc.org/projects/gretna/; used in Griffis et al., Cell eports, 2019)<br>- Analyze v.12 (https://analyzedirect.com/; used in Griffis et al., Cell eports, 2019)<br>- Surf Ice v2 (https://www.nitrc.org/projects/surfice; used in Griffis et al., Cell eports, 2019)<br>- MRIcroGL v1.2.2021 (https://www.nitrc.org/projects/mricrogl; used in Griffis et al., Cell eports, 2019)<br><br>Software used for the new analyses and results representation provided in this work:<br><br>- MATLAB 2021a (custum algorithms and specific toolboxes: Brain Connectivity Toolbox (https://sites.google.com/site/bctnet/); matlab_nifti (https://it.mathworks.com/matlabcentral/fileexchange/8797-tools-for-nifti-and-analyze-image?requestedDomain=); plotSpread (https://it.mathworks.com/matlabcentral/fileexchange/37105-plot-spread-points-beeswarm-plot); subperbar (http://mathworks.com/matlabcentral/fileexchange/57499-superbar); circulaGraph (https://github.com/paul-kassebaum-mathworks/circularGraph); BrainNet viewer (https://www.nitrc.org/projects/bnv/); simple_mixed_anova (https://www.researchgate.net/profile/Laurent-Caplette/publications); Violinplot (https://github.com/bastibe/Violinplot-Matlab))

- FSLeyes 0.34.2 (https://fsl.fmrib.ox.ac.uk/fsl/fslwiki/FSLeyes);

All custom algorithms used in this work are available at https://github.com/CorbettaLab/Favaretto2022NatComm. Correspondence related to the code should be addressed to C.F. (chiara.favaretto1990@gmail.com) or M.A. (michele.allegra@unipd.it).

For manuscripts utilizing custom algorithms or software that are central to the research but not yet described in published literature, software must be made available to editors and reviewers. We strongly encourage code deposition in a community repository (e.g. GitHub). See the Nature Portfolio guidelines for submitting code & software for further information.

Data

Policy information about availability of data

All manuscripts must include a data availability statement. This statement should provide the following information, where applicable:

- Accession codes, unique identifiers, or web links for publicly available datasets
- A description of any restrictions on data availability
- For clinical datasets or third party data, please ensure that the statement adheres to our policy

Source data to reproduce the main figures are provided with this paper. Raw neuroimaging and neuropsychological data are publicly available at https://cnda.wustl.edu/data/projects/CCIR_00299 and require controlled access as they contain sensitive patients' data. The person requesting the data must sign a confidentiality agreement provided by Washington University stipulating that they will make no attempt at identifying the patients and that they will use data for research purposes only. Correspondence and requests should be addressed to M.C. (maurizio.corbetta@unipd.it).

Field-specific reporting

Please select the one below that is the best fit for your research. If you are not sure, read the appropriate sections before making your selection.

☒ Life sciences ☐ Behavioural & social sciences ☐ Ecological, evolutionary & environmental sciences

For a reference copy of the document with all sections, see nature.com/documents/nr-reporting-summary-flat.pdf

Life sciences study design

All studies must disclose on these points even when the disclosure is negative.

Sample size

The sample size was determined based on previous works relating to the same dataset (e.g., Corbetta et al., Neuron, 2015; Siegel et al., PNAS, 2016; Griffis et al., Cell Reports, 2019).
Furthermore, we considered Leonardi et al. (Neuroimage, 2015) to ensure that the data were suitable to the dynamical analysis we performed.

Data exclusions

As a result of the pre-processing, 114 subjects were available at 2 weeks (sub-acute), 79 at 3 months, and 64 at 12 months, 24 and 22 controls at the first and second acquisition, respectively.
For the implementation of the dynamical functional analysis, only subjects with a sufficient number of good frames (300) were considered.

Patients:
We selected only patients, who participated to all the three recordings (2 weeks, 3 months, 12 months after stroke). Threfore, 47 patients were considered.
Controls:
The number of control subjects with sufficient frames were 20 during the first visit and 20 during the second visit. To avoid one group dominating the other in the following analysis steps, we equalized the number of controls and patients. Thus, we used all the controls' data as they were from different subjects.

Replication

Our results are based on a single experiment and have not been replicated in an independent cohort.

We controlled that our results did not depend on the choice of the parameters that we apply during the analysis. In particular the following test were performed:
- we verified that our results were not dependent on the fact that we considered all the controls' data as they were from different subjects. Specifically, all the analyses have been re-run considering the averaged measures over sections for the subset of control subjects, who participated to both sections.
- we tested the quality of the dimensionality reduction process: (1) test of the similarity of the reduced FC with the unreduced FC, and check that within-network connectivity was significantly stronger than between-networks connectivity. (2) verification that all previous results on stroke impairment in static FC were reproduced with the reduced data.
- we analyzed the impact of sliding windows width selection during the definition of the Dynamical Functional States (DFSs). From the results, we can confirm that our choice for the sliding window width did not impact our main results.

Randomization

Patients and control subjects were allocated in two different groups, based on clinical evaluation.

Blinding

The staff that was involved in segmenting or in reviewing the lesions was blind to the individual behavioral data.

Reporting for specific materials, systems and methods

We require information from authors about some types of materials, experimental systems and methods used in many studies. Here, indicate whether each material, system or method listed is relevant to your study. If you are not sure if a list item applies to your research, read the appropriate section before selecting a response.

Materials & experimental systems

n/a	Involved in the study
☒	☐ Antibodies
☒	☐ Eukaryotic cell lines
☒	☐ Palaeontology and archaeology
☒	☐ Animals and other organisms
☐	☒ Human research participants
☐	☒ Clinical data
☒	☐ Dual use research of concern

Methods

n/a	Involved in the study
☒	☐ ChIP-seq
☒	☐ Flow cytometry
☐	☒ MRI-based neuroimaging

Human research participants

Policy information about studies involving human research participants

Population characteristics

Subject demographics are described in detail in Corbetta M, et al. (2015) "Common behavioral clusters and subcortical anatomy in stroke". Neuron 85(5):927–941.

132 patients met all inclusion criteria and completed the entire subacute protocol (mean age 52.8 y with range 22–77; 119 right handed, 63 female, 64 right hemisphere).
31 controls completed the entire subacute protocol [mean age 55.7 y (SD = 11.5) with a range 21–83].

Recruitment

Subject enrollment is described in detail in Corbetta M, et al. (2015) "Common behavioral clusters and subcortical anatomy in stroke". Neuron 85(5):927–941.

Stroke patients:
Subjects (n = 172) were prospectively recruited, of whom 132 met post-enroll- ment inclusion criteria.
Inclusion Criteria: (1) Age 18 or greater. No upper age limit. (2) First symp- tomatic stroke, ischemic or hemorrhagic. (3) Up to two lacunes, clinically silent, less than 15 mm in size on CT scan. (4) Clinical evidence of motor, language, attention, visual, or memory deficits based on neurological examination. (5) Time of enrollment: < 2 weeks from stroke onset. (6) Awake, alert, and capable of participating in research.
Exclusion criteria: (1) Previous stroke based on clinical imaging. (2) Multi- focal strokes. (3) Inability to maintain wakefulness in the course of testing. (4) Presence of other neurological, psychiatric or medical conditions that preclude active participation in research and/or may alter the interpretation of the behavioral/imaging studies (e.g., dementia, schizophrenia), or limit life expectancy to less than 1 year (e.g., cancer or congestive heart failure class IV). (5) Report of claustrophobia or metal object in body.

Control subjects:
A healthy control group (n = 31) were matched with the study sample for age, gender, and years of education.

Ethics oversight

This research complies with all relevant ethical regulations. Written informed consent was obtained from all participants in accordance with the Declaration of Helsinki and procedures established by the Washington University in Saint Louis Institutional Review Board. All participants were compensated for their time. All aspects of this study were approved by the Washington University School of Medicine (WUSM) Internal Review Board.

Note that full information on the approval of the study protocol must also be provided in the manuscript.

Clinical data

Policy information about clinical studies
All manuscripts should comply with the ICMJEguidelines for publication of clinical research and a completedCONSORT checklist must be included with all submissions.

Clinical trial registration

NA

Study protocol

Study protocol is described in full detail in our previous publication (Corbetta et al., Neuron, 2015).

Data collection

Retrospective study. Data were collected using a Siemens 3T Tim-Trio scanner at the Washington University School of Medicine. Participants performed a behavioral battery consisting of multiple assessments within motor, language, attention, verbal memory, spatial memory, and visual domains, at the Washington University School of Medicine.

Outcomes

From Neuroimaging data, lesion identification and resting-state fMRI data have been directly derived. From lesion data, the disconnectome was derived as described in Griffis et al (Cell Reports, 2019). From fMRI data, we derived both static functional

connectivity through Pearson's correlation and dynamical connectivity measures as described below in the 'Models and Analysis' section.
From behavioral scores, principal components analyses (PCA) were used to decompose the behavioral data from each domain. Detailed descriptions of the behavioral testing and PCA analyses can be found in the Supplemental Material for Corbetta et al. (Neuron, 2015) and Siegel et al. (PNAS, 2016).

Magnetic resonance imaging

Experimental design

Design type: Resting-state

Design specifications: Six to eight resting state (RS) fMRI runs, each including 128 volumes (30 min total), for each subjects, and for each section.
Stroke patients' data were collected at three time points: 1-2 weeks after stroke, 3 months after stroke, 1 year after stroke.
Control subjects' data were recorded twice, 3 months apart.

Behavioral performance measures: NA

Acquisition

Imaging type(s): functional, structural

Field strength: 3 Tesla

Sequence & imaging parameters: Structural MRI:
(1) a sagittal T1-weighted MP-RAGE (TR = 1950 msec, TE = 2.26 msec, flip angle=90°, voxel size=1.0×1.0×1.0 mm); (2) a transverse T2-weighted turbo spin-echo (TR = 2500 msec, TE=435msec, voxel- size=1.0×1.0×1.0mm); and (3) sagittal FLAIR (fluid attenuated inversion recovery) (TR = 7500 msec, TE = 326 msec, voxel-size=1.5×1.5×1.5mm).

Functional MRI:
gradient echo EPI sequence (TR = 2000 msec, TE = 27 msec, 32 contiguous 4 mm slices, 4×4mm in-plane resolution) during which participants were instructed to fixate on a small white cross centered on a screen with a black background in a low luminance environment. Six to eight resting state (RS) fMRI runs, each including 128 volumes (30 min total), were acquired.

Area of acquisition: Whole brain scan

Diffusion MRI: ☐ Used ☒ Not used

Preprocessing

Preprocessing software: The complete preprocessing protocol is described in detail in Siegel et al, PNAS, 2016 and Griffis et al, Cell Reports, 2019.

Individual T1 MRI images were registered to the Montreal Neurological Institute brain using FSL (FMRIB Software Library) FNIRT (FMRIB nonlinear imaging registration tool) [Andersson JL, Jenkinson M, Smith S (2007) Non-linear optimisation. FMRIB technical report TR07JA1. Univ Oxf FMRIB Cent Oxf UK]. Lesions were manually segmented on individual structural MRI images (T1-weighted MP-RAGE, T2-weighted spin echo images, and FLAIR images obtained 1–3 wk poststroke) using the Analyze biomedical imaging software system (www. mayoclinic.org; Robb RA, Hanson DP (1991) A software system for interactive and quantitative visualization of multidimensional biomedical images. Australas Phys Eng Sci Med 14(1):9–30.). Two board-certified neurologists (M.C. and Alexandre Carter) reviewed all segmentations.

Normalization: Functional MRI data pre-processing consisted of slice-timing correction using sinc interpolation, correction of inter-slice intensity differences resulting from interleaved acquisition, normalization of whole-brain intensity values to a mode of 1000, correction for distortion via synthetic field map estimation, and within- and between- scan spatial re-alignment. BOLD data were re-aligned, coregistered to the corresponding structural images, normalized to atlas space, and resampled to 3mm cubic voxel resolution using a combination of linear transformations and non-linear warps.

Normalization template: MNI152

Noise and artifact removal: Processing steps were applied to account for non-neural sources of signal variance. Confounds related to head motion, global signal fluctuations, and non-gray matter signal compartments were removed from the data by regression of the six head motion parameters obtained from rigid body correction, along with the global GM signal and the CSF and white matter signals extracted from FreeSurfer tissue segmentations (Dale et al., Neuroimage, 1999). BOLD data were band-pass filtered ($0.009 < f < 0.08$ Hz) to retain low-frequency fluctuations.

Volume censoring: A frame was censored if it exceeded a 0.5 mm framewise displacement threshold, and the succeeding frame was also censored to further reduce confounds related to motion (Power et al., Neuroimage, 2014).

Statistical modeling & inference

Model type and settings	Dynamical functional analysis based on resting-state fMRI time series.
Effect(s) tested	Multiple measures ANOVA tests; Generalized Mixed Effect Linear Models; Nonparametric tests: Kruskal-Wallis test and Wilcoxon signed ank test.

Specify type of analysis: [x] Whole brain [ ] ROI-based [ ] Both

Statistic type for inference (See Eklund et al. 2016)	Parcel-wise analysis based on Gordon rsMRI parcellation (https://sites.wustl.edu/petersenschlaggarlab/resources/) and subcortical FreeSurfer parcellation. The original cortical parcellation includes 333 regions, but all regions with less than 20 vertices (approximately 50 mm^2) were excluded, as in previous works (Siegel et al., 2016, PNAS; Griffis et al. 2019, Cell Reports). Custum spatial clustering has been applied as described in the Supplementary Material of this work.
Correction	All statistical analyses have been corrected for multiple comparisons with FDR or Bonferroni.

Models & analysis

n/a	Involved in the study
[ ]	[x] Functional and/or effective connectivity
[ ]	[x] Graph analysis
[ ]	[x] Multivariate modeling or predictive analysis

Functional and/or effective connectivity

Static Functional Connectivity: z-Fisher tranform of Pearson's correlation
Dynamical Functional States: sliding-window temporal correlation (z-Fisher tranform of Pearson's correlation; window width=60s, window step=2s) followed by eigenvector decomposition and clustering (K-means)

Graph analysis

Newman's modularity: we used the code from the Brain Connectivity Toolbox, publicly available at sites https://sites.google.com/site/bctnet/, with modules' assignments chosen a priori based on Info-Map community detection in Gordon et al. 2016. Modularity was calculated at edge densities ranging from 4% to 20%, as suggested in Gratton et al 2012, without binarizing and with the symmetric treatment of negative weights. The average modularity across densities was used as final measure.
The measures were computed in each sliding window, and then sorted across dynamical functional states (DFSs).

Multivariate modeling and predictive analysis

Dynamical Principal Components Analysis:
In order to reduced the complexity of the whole set of dynamical measures describing how functional connectivity states evolve during time in control subjects and stroke ptients, a Principal Components Analysis (PCA) has been applied to all the dynamical measures (i.e. frequency of occurrence of each state, life-span of each state and transition probability between each pair of states). From this analysis a set of 3 dynamical principal components has resulted to explain the majority of variance across subjects. These multivariate measures have been used as input for the Ridge Regression analysis (see below).

Ridge Regression (RR) analysis:
dependent measure: dynamical principal components. These components were the results of a principal components analysis (PCA) applied to the all set of dynamical measures evaluated from the dynamics of each DFS for each subject (i.e. frequency of occurrence, life-span and transition probabilities). 3 dynamical components were derived and used as the dependent variable in the RR model (one at a time).
independent variables (predictors): we performed two different RR analyses:
1) we used the lesions mask (binary matrices, where each cell is equal to 1 if the related voxel is lesioned, and equal to 0 otherwise) as predictors
2) we used the weighted disconnectomes as predictors (weighted matrices, where each cell represents the percentage of disconnected streamlines of a specific pair of regions.
In both cases, we applied a PCA to the regressors before appliyng the RR models.
The significance level of each results was derive thorugh a permutations analysis (10,000 permutations).
